# Efficacy of pharmacotherapeutics for patients comorbid with alcohol use disorders and depressive symptoms—A bayesian network meta‐analysis

**DOI:** 10.1111/cns.13437

**Published:** 2020-07-19

**Authors:** Jiande Li, Hongxuan Wang, Mei Li, Qingyu Shen, Xiangpen Li, Xiaoming Rong, Ying Peng

**Affiliations:** ^1^ Department of Neurology Sun Yat‐Sen Memorial Hospital Sun Yat‐Sen University Guangzhou China; ^2^ Guangdong Provincial Key Laboratory of Malignant Tumour Epigenetics and Gene Regulation Sun Yat‐Sen Memorial Hospital Sun Yat‐Sen University Guangzhou China

**Keywords:** alcohol use disorders, AUD, depression, depressive symptoms, drug, therapy

## Abstract

**Background:**

We aimed to compare and rank the efficacy of different pharmacotherapeutics for patients comorbid with alcohol use disorders and depressive symptoms.

**Method:**

Bayesian network meta‐analysis was performed for three different outcome parameters: alcohol use disorders (AUD) remission rate, percent abstinent days, and scores of depression scales. The surface under the cumulative ranking curves (SUCRA) was used for ranking the efficacy of interventions. Sensitivity analysis and direct pairwise analysis were conducted to validate the main results.

**Results:**

A total of 68 RCTs consisting of 5890 patients were included. Disulfiram could significantly increase the AUD remission rates (OR 5.02, 1.97‐12.95) and the percent abstinent days (MD 17.08, 3.48‐30.93). Disulfiram was associated with the best efficacy in achieving remission (SUCRA 95.1%) and increasing abstinent days (SUCRA 87.6%). Noradrenaline reuptake inhibitor was significantly more efficacious than controls (SMD −2.44, −3.53 to −1.36) and have the first rank (SUCRA 99.0%) in reducing the scores of depression scales. Antiepileptics have relatively higher ranks in efficacy for both AUD and depressive symptoms.

**Conclusions:**

Disulfiram was associated with the best efficacy in achieving abstinence for comorbidity patients. Noradrenaline reuptake inhibitor was demonstrated to be associated with the best efficacy in reducing scores of depression scales. Antiepileptics might be beneficial to both alcohol‐related and depressive symptoms.

## Graphical abstract

The present study conducts a Bayesian network meta‐analysis to compare the efficacy of different pharmacotherapies for patents comorbid with alcohol use disorders and depressive symptoms. Disulfiram was associated with the best efficacy in achieving abstinence, noradrenaline reuptake inhibitor was demonstrated to be the best choice to reduce the scores of depression scales, and antiepileptics might be beneficial to both alcohol‐related and depressive symptoms.

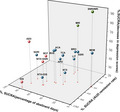

## 1 INTRODUCTION

Alcohol use disorders (AUD), including alcohol abuse and alcohol dependence, are common psychiatric disorders contribute greatly to the burden of society.^1^ Alcohol‐related diseases are one of the leading risk factors of disability and mortality, causing about 3.3 million deaths around the world and taking account for 5.1% of the global burden of disease according to the report of WHO.^2^

Depression is a highly prevalent mental illness and is one of the important causes of premature deaths. About 350 million people around the world suffer from depression and nearly one million of them commit suicide every year.^3^ Depression will become the second leading cause of global disease burden by 2030.^4^

According to the results of epidemiological studies, alcohol use disorders and depression often comorbid with each other.^5^, ^6^, ^7^ It was suggested that suffering from one disease would double the risk of the other.^8^ Evidence from longitudinal studies also indicates that there might be a causal link between alcohol use disorders and depression.^9^, ^10^

The comorbidity of the two diseases can aggravate the condition and worsen the prognosis. On one hand, the existence of alcohol use disorders would prolong the duration of depression, leading to more frequent depressive episodes, and a higher risk of suicide.^11^, ^12^ On the other hand, lingering depression increases not only the mood‐induced episodes of heavy drinking, but also the risk of relapse during the early abstinence.^13^, ^14^ Therefore, the treatment and management of both AUD and depression are important public health issues.

Although there are approved medications for AUD and depression alone, the efficacy of these medications in patients comorbid with two diseases is still unclear. Randomized controlled trials (RCTs) of various pharmacotherapy including anti‐craving drugs,^15^, ^16^, ^17^, ^18^, ^19^ antidepressants,^20^, ^21^, ^22^, ^23^, ^24^ antiepileptics,^25^, ^26^, ^27^, ^28^, ^29^ and antipsychotics^30^, ^31^, ^32^ have drawn inconsistent results. Moreover, in clinical practice, it is a challenge in making a decision when facing various available medication options. Therefore, this study aimed at comparing and ranking the efficacy of various pharmaceutic options in treating patients comorbid with alcohol use disorders and depressive symptoms by conducting a Bayesian network meta‐analysis.

## 2 METHOD

### 2.1 Search strategy

This study was performed according to the Preferred Reporting Items for Systematic Reviews and Meta‐analyses (PRISMA) statement extension for network meta‐analysis.^33^ We conducted a comprehensive search in PubMed, Embase, Cochrane CENTRAL, PsycINFO, Cochrane Drugs and Alcohol Group (CDAG), and The Cumulative Index to Nursing and Allied Health Literature (CINAHL) database from inception to January 15, 2020. Construction of search strategy was based on MeSH terms plus free texts. Table S1 presents an example of a search strategy used in PubMed. We also conducted a supplementary search by reviewing the reference lists of related studies and searching related studies in ClinicalTrial.gov.

### 2.2 Study selection

Randomized clinical trials (RCTs) that compared the efficacy of pharmaceutic interventions with placebo or no‐treatment control or with each other for adults comorbid with alcohol use disorders and depression or depressive symptoms were considered eligible. In the current study, alcohol use disorders should be ascertained based on DSM or ICD‐10 diagnostic criteria. Depression should be ascertained through DSM or ICD‐10 diagnostic criteria. Besides, to fully represent the situation encountered by physicians in clinical practice, study with patients scoring more than the cutoff threshold of valid depression symptoms scales (eg, score more than 7 in the Hamilton Depression Scale) was also considered eligible.

In the current study, outcomes of interest for AUD were AUD remission rate (ie, the percentage of patients who remain consistent abstinence or don't relapse into heavy drinking during the whole treatment session) and percent of abstinent days. Because the measurement of abstinence and no relapse into heavy drinking is less likely to be affected by recall bias than the measurement of alcohol consumption,^34^ the outcome of interest for depressive symptoms was the endpoint scores of the depressive symptom scales.

Studies met one or more of the following criteria were excluded: (a) not RCTs (eg, observational studies, reviews, comments, and case reports); (b) participants were mainly adolescents; (c) studies regarded acute alcohol withdrawal; (d) participants comorbid psychosis such as bipolar disorders, schizophrenia, et al.; (e) participants comorbid severe internal disease such as heart failure, liver cirrhosis et al.; (f) interventions belong to the same type; (g) no appropriate outcomes; (h) studies based on the same research; (i) studies compared pharmacotherapy with psychotherapy. Two investigators independently selected the studies, and a third investigator was consulted to resolve controversies.

### 2.3 Data extraction and quality assessment

We used a pre‐set collection form to extract basic information, intervention characteristics, and outcome data. We preferred to extract the data of treatment sessions. When data of the intention‐to‐treat sample and data of completers were both available, the former was preferable. For those studies with only survival curves available, we used Getdata (version 2.2) to extract the raw data. Two investigators independently conducted the data extraction, and any contradictions were resolved by discussion or by consulting with a third investigator. We used the tool advised by the Cochrane Handbook to assess the risk of bias and evaluate the quality of each included study.^35^ The graphics of the summary of the overall risk of bias and the study‐level risk of bias were conducted using Review Manager Software (version 5.3).

### 2.4 Statistical analysis

We conducted the network meta‐analysis based on the Bayesian method that is more flexible in modeling and more accurate in pooling the results than the traditional frequency theory method.^36^, ^37^ Network plot was portrayed to present the comparison network of interventions and controls. Nodes represented interventions/controls, and lines represented direct comparisons. The size of nodes represents the size of included sample, and the width of lines represents the number of included trails. The value of deviance information criterion (DIC) was used to determine whether to use a random model or a fixed model. The smaller the DIC value, the better the model fit. The Markov‐chain Monte Carlo simulation (MCMC) method was applied. The number of simulation chains is four, and the number of tuning iteration and simulation iterations was 50 000 and 200 000, respectively. Brooks‐Gelman‐Rubin method was used to assess the iterative simulation.^38^ The surface under cumulative ranking curves (SUCRA) values were used for hierarchically ranking the efficacy of interventions. The more a SUCRA value is approached to 100%, the more the corresponding intervention is likely to achieve the best treatment efficacy.^39^, ^40^ Odds ratios (ORs) were used for binary data (ie, AUD remission rate), and mean differences (MDs) were used for continuous data. Standard mean differences (SMDs) were applied for the situation where different measurement tools were applied among included studies. The node‐splitting analysis was used to assess the local inconsistency, a *P*‐value > .05 indicated no significant inconsistency between the direct pairwise results and the indirect results.^41^ A consistency model is preferred if there is no significant inconsistency indicated. Publication bias was assessed using funnel plots.

Sensitivity analyses were conducted by only including studies that had a treatment session no less than 8 weeks and among studies involved participants with at least moderate depressive symptoms (assessed by valid scales) or with a diagnosis of depression (ascertained by standard criteria) to corroborate the main results.

We also performed direct pairwise analysis by comparing the efficacy of the top five therapeutics (ascertained through SUCRA value) with their corresponding lower‐ranked therapeutics for both AUD and depression outcome parameters. Moreover, since previous traditional meta‐analysis drew inconsistent results on whether antidepressants confer any benefit to improving depressive symptoms compared to controls.^42^, ^43^ Therefore, we compared the efficacy of non‐antidepressant and antidepressant with controls in improving depressive symptoms, respectively. In this case, heterogeneity of pairwise analysis was assessed with *I* ^2^ statistics, in which an *I* ^2^ less than 50% indicated no significant heterogeneity.

Statistical analyses of Bayesian network meta‐analysis were conducted using the R software (version 3.6.2) and OpenBUGS software (version 3.2.3). Network plots, cumulative probabilities ranking plots, and pairwise analysis were conducted in Stata software (version 15.2).

## 3 RESULTS

**FIGURE 1:**
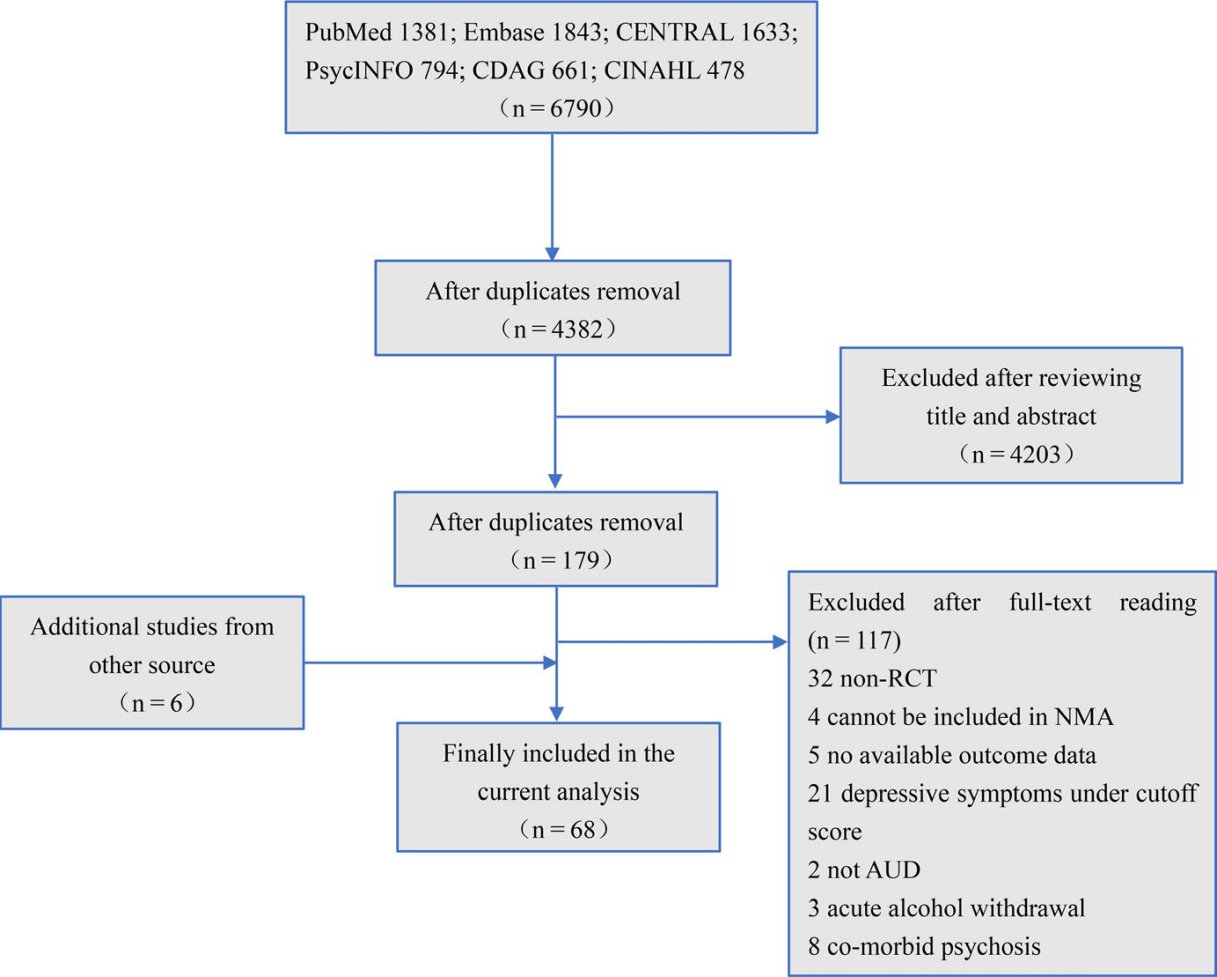
Flow chart of studies selection procedure and reasons for exclusion from the network meta‐analysis

We identified 6790 hits through searching databases, of which 4203 were removed after reviewing the titles and abstracts. Among the 179 studies included for full‐text reading, 117 of which were further excluded for various reasons. With five additional studies found from the references lists and one study found from ClinicalTrials.gov, eventually, 68 studies were included in the current analysis. A flow chart of studies selection was shown in Figure 1.

A large part of the included studies were two‐arm trials (85.3%), and the rest of them were three‐arm (11.8%) or four‐arm (3%) trials. We subdivided the included interventions into 18 categories: acamprosate, baclofen, disulfiram, naltrexone, buspirone, bromocriptine, lithium, memantine, antiepileptics (included carbamazepine, topiramate, and tiagabine here), antipsychotics (included aripiprazole and olanzapine here), selective serotonin reuptake inhibitor (SSRI, included citalopram, escitalopram, fluoxetine, paroxetine, and sertraline here), noradrenaline reuptake inhibitor (NRI, included venlafaxine and viloxazine here), serotonin receptor antagonist/reuptake inhibitor (SARI, included nefazodone and trazodone here), tricyclic antidepressants (TCA, included amitriptyline, imipramine, and desipramine here); noradrenaline and specific serotonin antagonist (NaSSA, included mirtazapine here); naltrexone plus disulfiram, and naltrexone plus SSRI. The 68 included RCTs ranged from 8 to 492 in sample sizes and totally consisting of 5891 cases. These studies published from 1976 to 2018 and were conducted in different countries, but most of them were in developed countries. The characteristics of the included studies were summarized in Table S2. The random effect model is adopted in this study for a smaller DIC value compared to the fixed effect model.

### 3.1 Effect of pharmacotherapies on the AUD remission rate

**FIGURE 2:**
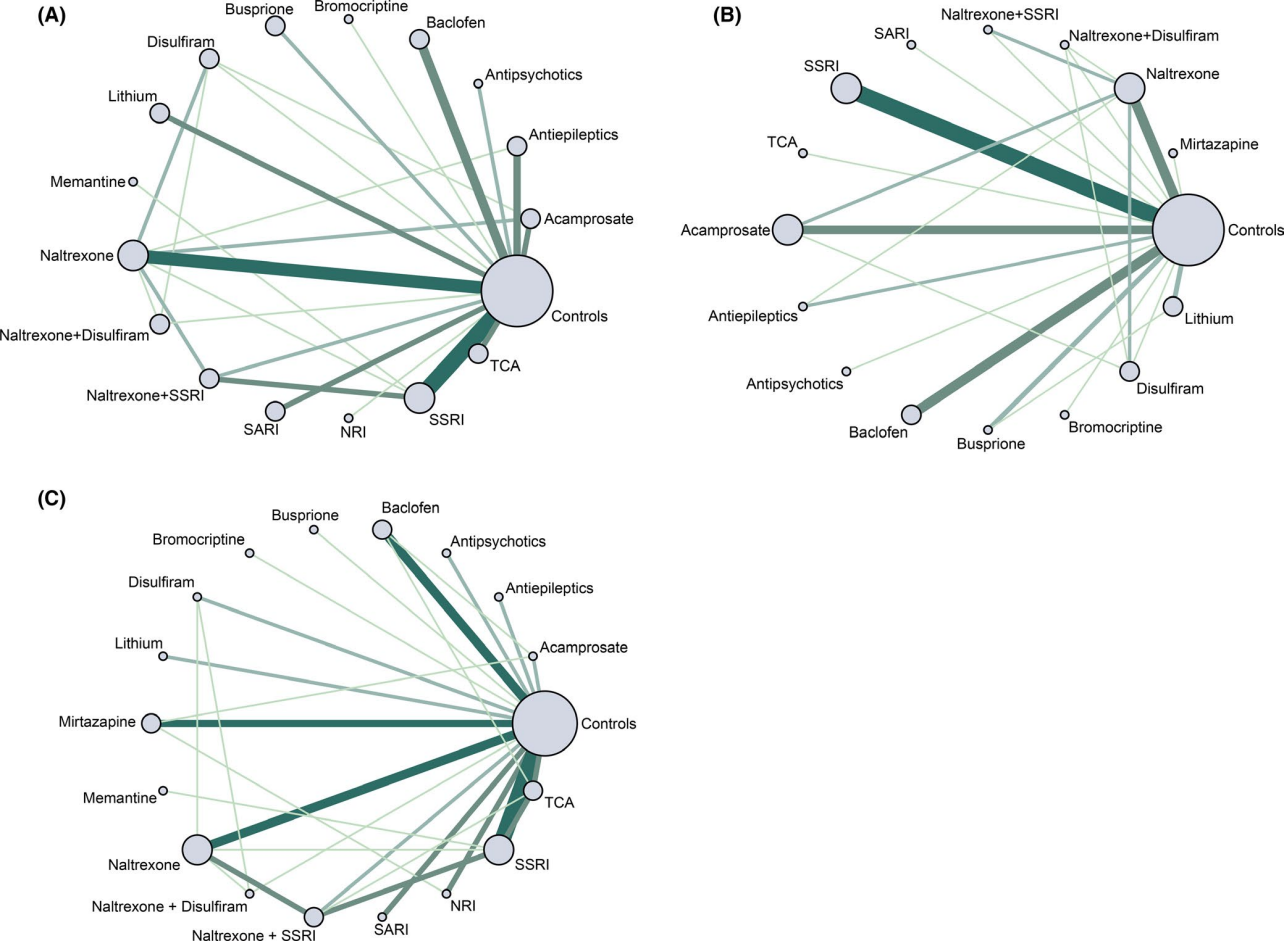
A, network plot for AUD remission; B, network plot for percent abstinent days; C, network plot for reduction of scores of validated depression scales. The size of nodes represents the relative size of included sample; the width of line represents the number of included trails. NRI, noradrenaline reuptake inhibitor; SARI, serotonin receptor antagonist/reuptake inhibitor; SSRI, selective serotonin reuptake inhibitor; TCA, tricyclic antidepressants

**FIGURE 3:**
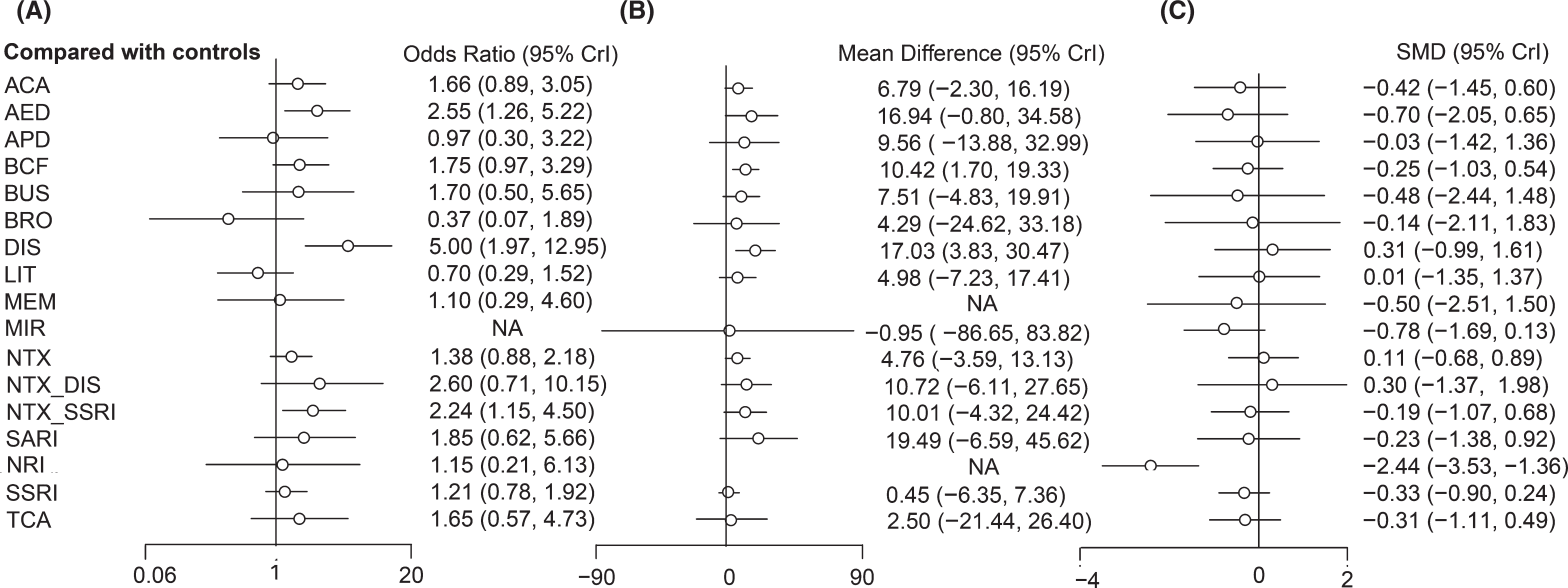
Forest plot of different interventions versus controls for A, AUD remission rate; B, percent abstinent days; C, reduction in scores of depression scales. SMD, standard mean difference. ACA NRI, noradrenaline reuptake inhibitor; acamprosate; AED NRI, noradrenaline reuptake inhibitor; antiepileptics; APD, antipsychotics; BCF, baclofen; BRO, bromocriptine; BUS, buspirone; DIS, disulfiram; LIT, lithium; MEM, memantine; MIR, mirtazapine; NRI, noradrenaline reuptake inhibitor/noradrenaline reuptake inhibitor; NTX, naltrexone; SSRI, selective serotonin reuptake inhibitor; SARI, serotonin receptor antagonist/reuptake inhibitor; TCA, tricyclic antidepressants

**FIGURE 4:**
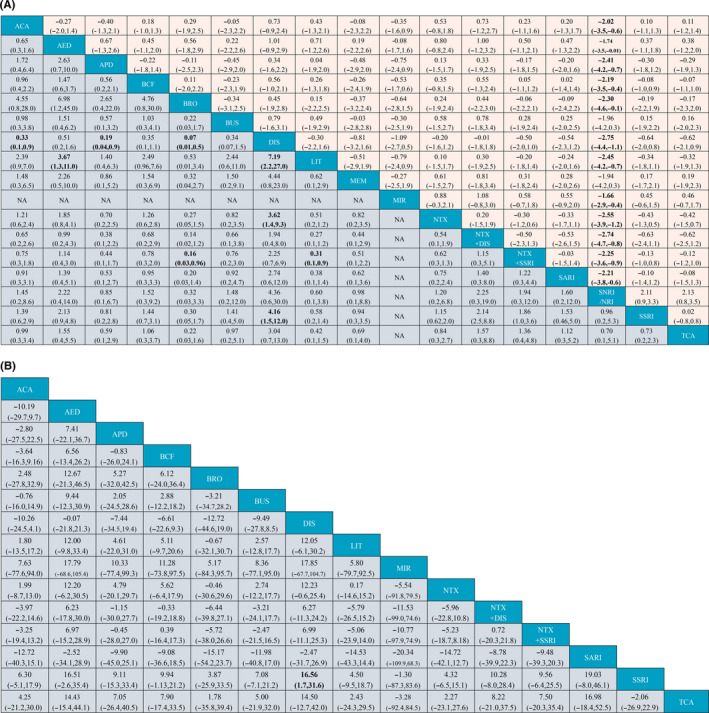
A, League table of comparisons between all interventions. Estimates represent ORs and 95% CrI of the AUD remission rate (purple region) and SMDs and 95% CrI of the reduction in scores of depression scales (orange region), respectively. Each estimate was pooled from the comparison between a column intervention and a row intervention. ACA, acamprosate; AED, antiepileptics; APD, antipsychotics; BCF, baclofen; BRO, bromocriptine; BUS, buspirone; DIS, disulfiram; LIT, lithium; MEM, memantine; MIR, mirtazapine; NRI, noradrenaline reuptake inhibitor/noradrenaline reuptake inhibitor; NTX, naltrexone; SARI, serotonin receptor antagonist/reuptake inhibitor; SSRI, selective serotonin reuptake inhibitor; TCA, tricyclic antidepressants. B, League table of comparisons between all interventions. Estimates represent MDs and 95% CrI of the reduction in scores of depression scales (purple region). Each estimate was pooled from the comparison between a column intervention and a row intervention. ACA, acamprosate; AED, antiepileptics; APD, antipsychotics; BCF, baclofen; BRO, bromocriptine; BUS, buspirone; DIS, disulfiram; LIT, lithium; MEM, memantine; MIR, mirtazapine; NRI, noradrenaline reuptake inhibitor/noradrenaline reuptake inhibitor; NTX, naltrexone; SSRI, selective serotonin reuptake inhibitor; SARI, serotonin receptor antagonist/reuptake inhibitor; TCA, tricyclic antidepressants

**FIGURE 5:**
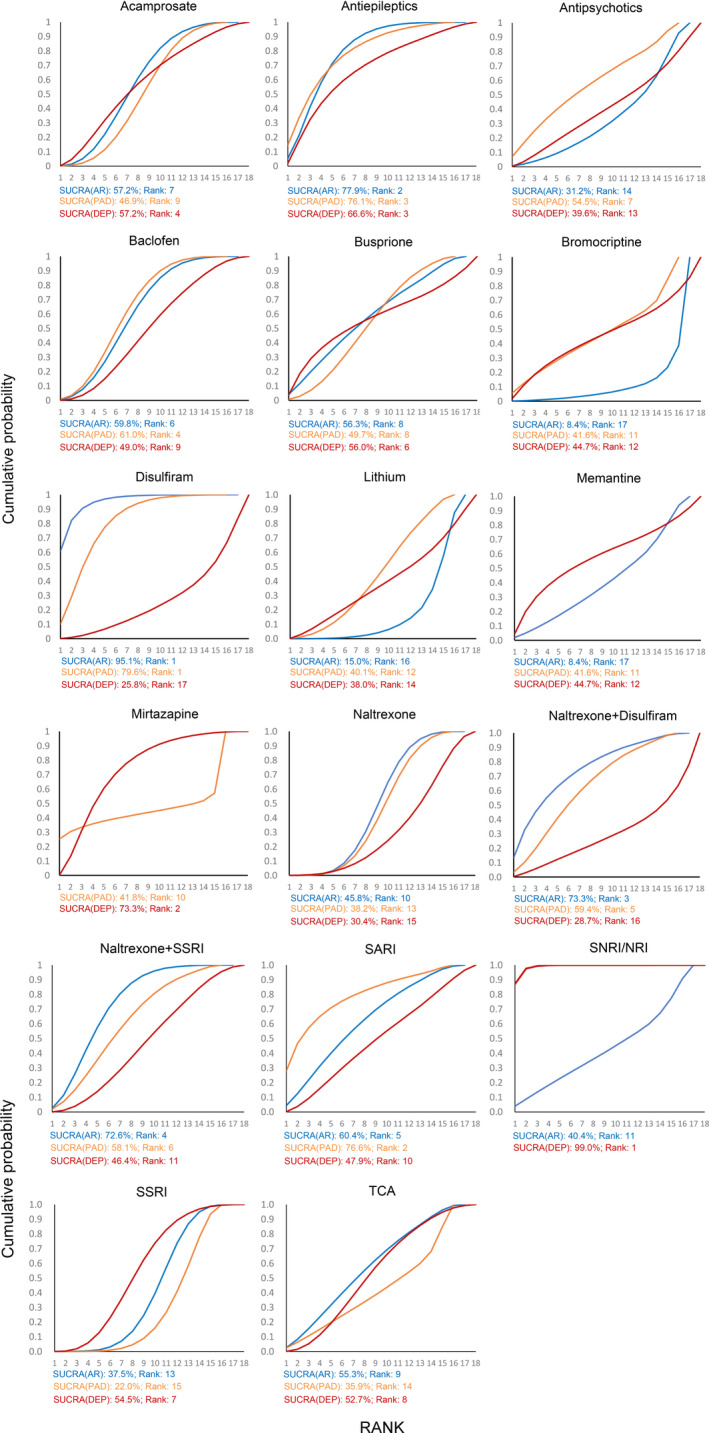
Cumulative probability ranking curve of different interventions for three outcome parameters: AR, AUD remission rate (blue curve); PAD, percent abstinent days (orange curve); DEP, reduction in scores of depression scales (red curve). The more a surface under the cumulative ranking curve (SUCRA) value approached to 100%, the better the corresponding intervention is in efficacy

**FIGURE 6:**
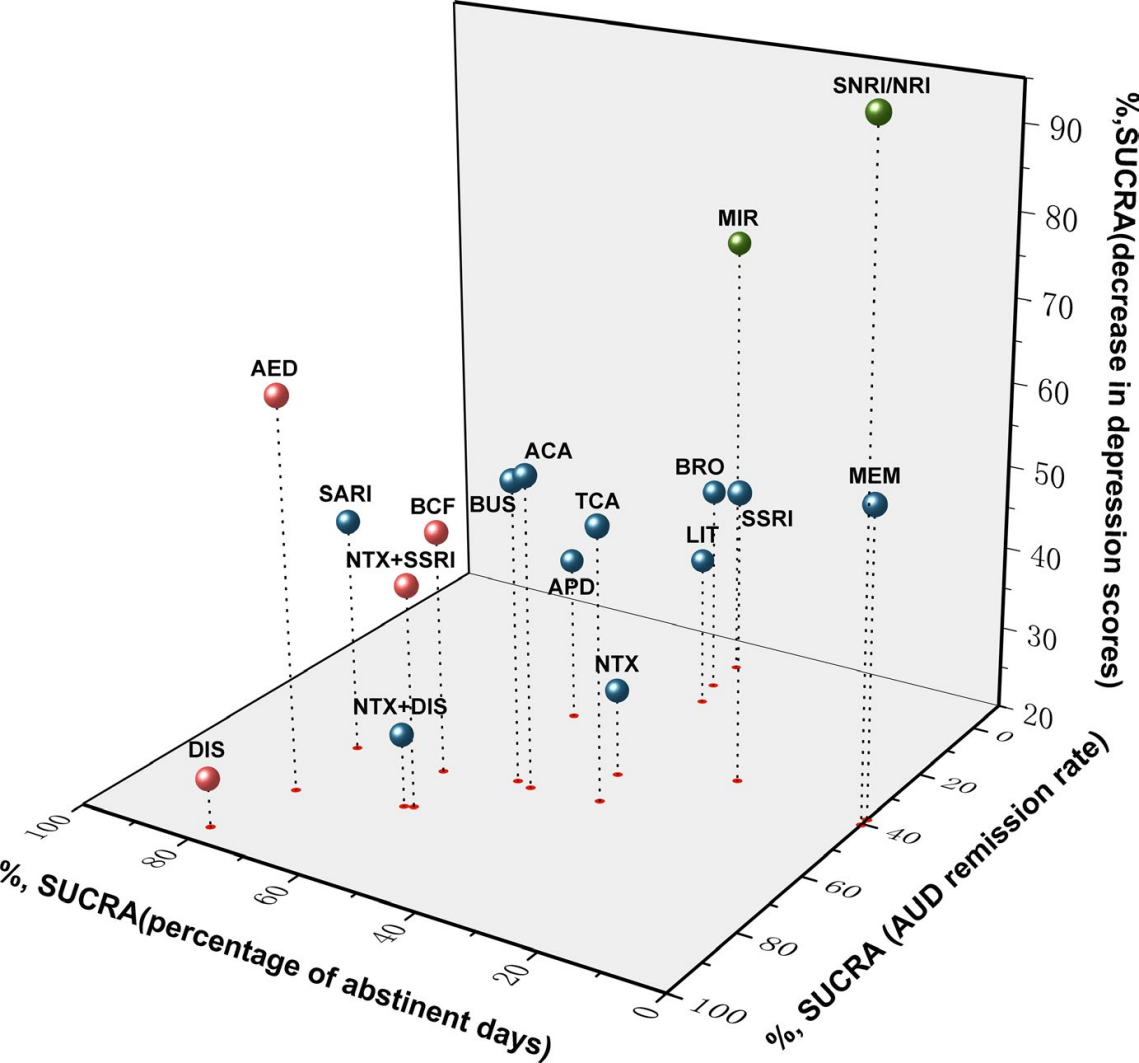
Three‐dimension scatters plot for SUCRA value for three different parameter outcomes. The more a SUCRA value approached to 100%, the better the corresponding intervention is in efficacy. Red colored represented an intervention was significantly more efficacious in the treatment of alcohol‐related symptoms compared to controls. Green colored represented an intervention was significantly more efficacious in reducing the scores of depression scales. ACA, acamprosate; AED, antiepileptics; APD, antipsychotics; BCF, baclofen; BUS, buspirone; BRO, bromocriptine; DIS, disulfiram; LIT, lithium; MEM, memantine; MIR, mirtazapine; NRI, noradrenaline reuptake inhibitor; NTX, naltrexone; SARI, serotonin receptor antagonist/reuptake inhibitor; SSRI, selective serotonin reuptake inhibitor; TCA, tricyclic antidepressants

For AUD remission rates, 44 trials^15^, ^16^, ^17^, ^18^, ^19^, ^21^, ^22^, ^23^, ^24^, ^25^, ^27^, ^28^, ^29^, ^30^, ^31^, ^44^, ^45^, ^46^, ^47^, ^48^, ^49^, ^50^, ^51^, ^52^, ^53^, ^54^, ^55^, ^56^, ^57^, ^58^, ^59^, ^60^, ^61^, ^62^, ^63^, ^64^, ^65^, ^66^, ^67^, ^68^, ^69^, ^70^, ^71^, ^72^ consisting of 4334 cases were included in analysis. Comparisons between 16 different pharmacotherapeutic options and controls were portrayed in a network plot (Figure 2A). Among the 25 direct pairwise comparisons, 11 of which regarded the comparison between different pharmacotherapeutics options. A large proportion of included studies regarded the comparison between baclofen, naltrexone, SSRI, and controls. Disulfiram (OR 5.02, 1.97‐12.95), antiepileptics (OR 2.55, 1.26‐5.22), and naltrexone plus SSRI (OR 2.24, 1.15‐4.50) were significantly better than controls in increasing the AUD remission rate (Figure 3A). Disulfiram was significantly more efficient than acamprosate, antipsychotics, bromocriptine, lithium, naltrexone, and SSRI in achieving AUD remission (Figure 4A). Besides, based on the results of cumulative probabilities ranking (Figures 5 and 6), disulfiram possessed the best rank in the efficacy of achieving AUD remission (SUCRA 95.1%), followed by antiepileptics (SUCRA 77.9%), naltrexone plus disulfiram (SUCRA 73.3%), and naltrexone plus SSRI (SUCRA 72.6%). No significant publication bias was indicated by visually inspecting the funnel plot (Figure S1A). According to the node‐splitting analysis, there was no significant inconsistency detected between the results of the direct and indirect comparison among the 11 closed intervention loops (Figure S2A). The potential scale reduction factor (PSRF) was equal to one, indicating that the number of iterative simulations is enough to reach a good convergency.

In sensitivity analysis, after excluding 11 studies in which participants had only mild depressive symptoms, results indicated that disulfiram (OR 4.33, 1.14 to 17.51) and naltrexone plus SSRI (OR 2.55, 1.24 to 5.37) remained significantly better than controls in increasing the AUD remission rate (Table S3). Disulfiram still ranked the first according to the SUCRA value (87.6%). We also conducted a sensitivity analysis by only including studies with treatment session ≥ 8 weeks, and results were similar to the main results (Table S4).

In pairwise analysis used for validation, the relatively higher‐ranked interventions, disulfiram, epileptics, and naltrexone plus SSRI could significantly increase the AUD remission rate compared to their corresponding lower‐ranked interventions, which corroborated the robustness of the main results (Table S5A).

### 3.2 Effect of pharmacotherapies on the percent abstinent days

As for percent abstinent days, a total of 39 studies^15^, ^16^, ^17^, ^18^, ^19^, ^21^, ^23^, ^24^, ^25^, ^26^, ^30^, ^44^, ^45^, ^46^, ^48^, ^49^, ^51^, ^52^, ^53^, ^55^, ^56^, ^58^, ^59^, ^60^, ^61^, ^62^, ^65^, ^67^, ^68^, ^73^, ^74^, ^75^, ^76^, ^77^, ^78^, ^79^, ^80^, ^81^, ^82^ consisting of 4050 cases and 15 different interventions were included in the analysis (Figure 2B). Among the 23 direct pairwise comparisons, 8 of which regarded the comparison between different pharmacotherapeutics options. A large part of included studies regarded the comparison between acamprosate, baclofen, naltrexone, SSRI, and controls. Disulfiram (MD 17.03, 3.83‐30.47) and baclofen (MD 10.42, 1.70‐19.33) were significantly associated with higher percentages of abstinent days during the treatment sessions (Figure 3B). Results of the comparisons between all interventions were shown in Figure 4B. Concerning the cumulative probabilities ranking (Figures 5 and 6), disulfiram had the largest SUCRA value (79.6%) in terms of increasing the abstinent days, followed by SARI (SUCRA 76.6%), antiepileptics (SUCRA 76.1%), and baclofen (SUCRA 61.0%). No significant publication bias was indicated by visually inspecting the funnel plot (Figure S1B). In the node‐splitting analysis (Figure S2B), inconsistency was detected in the comparison between disulfiram‐acamprosate (*P* = .03) and between disulfiram‐controls (*P* = .02). However, there was no significant inconsistency detected in the other seven closed loops. Besides, the DIC values of the consistency model (161.9) and the inconsistency model (160.5) were similar, and the p‐value of inconsistency model was 0.87. All of these indicated that the integral consistency was moderate. As for model convergency, the PSRF value was 1, indicating a good convergency.

In sensitivity analysis where only studies with participants having at least moderate depressive symptoms or having depression diagnosis were included, results from 28 studies showed that no intervention revealed significantly better efficacy than controls in increasing the abstinent days (Table S3). Sensitivity analysis was also conducted by excluding two studies^44^, ^59^ with treatment sessions less than 8 weeks. Results showed that only disulfiram was significantly better than controls in increasing the percentages of abstinent days (MD 17.08, 3.48 to 30.93) (Table S4).

In pairwise analysis used for validation, interventions with higher SUCRA (disulfiram, SARI, antiepileptics, baclofen, and naltrexone plus disulfiram) revealed better efficacy on increasing the percent abstinent days compared to their corresponding lower‐ranked interventions (Table S5B).

### 3.3 Effect of pharmacotherapies on the scores of depression scales

A total of 47 studies^15^, ^16^, ^18^, ^19^, ^20^, ^21^, ^22^, ^23^, ^24^, ^27^, ^28^, ^30^, ^31^, ^47^, ^48^, ^49^, ^50^, ^51^, ^52^, ^53^, ^54^, ^58^, ^60^, ^61^, ^63^, ^65^, ^69^, ^70^, ^72^, ^73^, ^76^, ^79^, ^80^, ^81^, ^82^, ^83^, ^84^, ^85^, ^86^, ^87^, ^88^, ^89^, ^90^, ^91^, ^92^, ^93^, ^94^ consisting of 3835 cases and 17 interventions were included in the analysis (Figure 2C). Among the 29 direct pairwise comparisons, 13 of which regarded the comparison between different pharmacotherapeutics options. A large part of included studies regarded the comparison between baclofen, naltrexone, SSRI, and controls. According to the network meta‐analysis, only NRI was significantly better than controls in reducing the endpoint scores of depression scale (SMD −2.44, −3.53 to −1.36, Figure 3C). Besides, NRI was significantly more efficient in improving depressive symptoms than other interventions except for memantine and buspirone (Figure 4A). Results of cumulative probabilities ranking (Figures 5 and 6) indicated that NRI was associated with the lowest scores of depression scales at the end of studies (SUCRA 99.0%), followed by mirtazapine (SUCRA 73.3%), antiepileptics (SUCRA 66.6%), and acamprosate (SUCRA 57.2%). Publication bias might exist since asymmetry was noticed by visually inspecting the funnel plot (Figure S1C). In the node‐splitting analysis, no significant inconsistency was detected (Figure S2C).

In sensitivity analysis, after excluding nine studies^15^, ^47^, ^50^, ^52^, ^53^, ^54^, ^72^, ^91^, ^94^ in which participants had only mild depressive symptoms, in addition to NRI (SMD −3.60, −4.91 to −2.29), mirtazapine was also demonstrated to be better than controls in reducing the scores of depression scales (SMD −0.99, −1.91 to −0.07) (Table S3). Results of ranking probabilities were similar to the main analysis with NRI (SUCRA 99.9%) still the first rank followed by mirtazapine (SUCRA 76.0%). We also conducted a sensitivity analysis of 39 studies that had treatment sessions no less than 8 weeks. Results showed that NRI still had the highest SUCRA (99.9%) and significantly better efficacy than controls (SMD −3.96, −5.33 to −2.59) (Table S4).

In the direct meta‐analysis, our findings indicated that compared to controls, pharmacotherapy could significantly reduce the scores of depression scales with a quite modest effect size (SMD −0.25, −0.41 to −0.09). However, only the subgroup of antidepressants (SMD −0.43, −0.68 to −0.17) revealed a significant effect size while thenon‐antidepressants subgroup (SMD −0.10, −0.31 to 0.11) did not (Figure S3A). Results remained similar after excluding one study^20^ that contributed to the heterogeneity (Figure S3B). In pairwise analysis compared the efficacy of higher‐ranked interventions to the lower ones, interestedly, NRI revealed no significant differences in reducing the scores of depression scales. We further divided NRI into viloxazine (selective noradrenaline reuptake inhibitor) and venlafaxine (serotonin and noradrenaline reuptake inhibitor), results indicated that only subgroup of viloxazine showed a benefit to reducing the scores of depression scales (Table S5C).

### 3.4 Quality of studies

As for quality assessment, 28 trials (41.2%) mentioned the detail about random sequence generation. 55 studies (80.9%) applied blinding methods but only 27 trials (39.7%) described allocation concealment. A large part of the included studies had a low risk of attrition bias. Overall, included studies were considered to be low to moderate in quality. The overall and study‐level risk of bias were summarized in Figure S4.

## 4 DISCUSSION

The current Bayesian network meta‐analysis included 5891 comorbidity patients across 68 studies. Our findings indicated that disulfiram, antiepileptics, baclofen, and naltrexone plus SSRI were significantly associated with higher AUD remission rates and (or) higher percent abstinent days during the treatment sessions when compared to controls. Among them, disulfiram possessed the best rank in efficacy. As for the outcome parameter for depressive symptoms, noradrenaline reuptake inhibitor (NRI) and mirtazapine exhibited statistical significance over controls in reducing the scores of depression. And NRI might be hierarchically the best in achieving the most pronounced reduction. However, the results should be interpreted cautiously since the significance of the effect size was limited to only selective noradrenaline reuptake inhibitor but not serotonin and noradrenaline reuptake inhibitor (SNRI) and potential publication might exist. Although not approved for the treatment of AUD or mono‐depression, antiepileptics might be a beneficial therapeutic option to both AUD‐related and depressive symptoms in comorbidity patients.

Our findings were consistent with some opinions of a recent systematic review^95^ which also concerned the treatment of patients comorbid with alcohol use disorders and depression. However, our study provided more information because we compared the efficacy of different interventions quantitatively using Bayesian network meta‐analysis and drew the results for different outcome parameters.

As one of the earliest approval medications for alcoholism, in our study, disulfiram was demonstrated to be the most efficacious pharmacotherapy in maintaining remission and increasing abstinence days for comorbidity patients. The results remained significant after taking into account the length of treatment sessions and the severity of depression, which corroborating the robustness of our findings. Although inconsistency was detected in comparison between controls, acamprosate, and disulfiram for percent abstinent days, which might be accounted for by the limited number of included studies, the benefit of disulfiram on achieving abstinence was supported by main results and pairwise analysis. Findings from a recent open‐label trial including 41 AUD patients were partly consistent with ours. It found that disulfiram plus lorazepam could significantly increase the percent abstinent days and reduce depressive symptoms.^96^ However, in our study, disulfiram showed no benefit to depressive symptoms compared to controls, indicating the need for combination with medication specifically targeting depressive symptoms. By extendedly blocking the acetaldehyde dehydrogenase, disulfiram could trigger uncomfortable "Antabuse" reactions such as tachycardia, flushing, and nausea after drinking, which gradually established the aversion to alcohol and finally achieve the aim of quitting drinking. In addition to the peripheral effect, disulfiram has also been shown to modulate the transmission of dopamine in central nervous systems that plays a pivotal role in reward circuit and substance dependence.^97^, ^98^

Another anti‐craving medication, the gamma‐aminobutyric acid (GABA)_B_ receptor agonist baclofen, exhibited statistical significance over controls and had a relatively higher rank of efficacy in increasing the percent abstinent days. However, the significance of effect size disappeared when the analysis was conducted only among studies with longer treatment phase and with more severe depression patients, which prevented broad interpretations. Other anti‐craving medication, including acamprosate and naltrexone, conferred no significant benefit to both AUD and depressive symptoms for comorbidity patients compared to controls, which is in line with the results from a recent systematic review.^95^ Another meta‐analysis also concluded that, in comparison with placebo, the number needed to treat (NNT) for acamprosate to prevent a return to any drinking and NNT for naltrexone to prevent a return to heavy drinking were both 12,^99^ indicating their limited pharmaceutic effect on achieving abstinence.

Antidepressants were supposed to be a pivotal part of treatment for patients with dual diagnosis since treatment targeted only alcohol‐related symptoms was insufficient to achieve complete remission of depression.^100^ However, previous meta‐analysis focus on the efficacy of antidepressants drew inconsistent results. Meta‐analysis performed by Foulds et al^101^ involved 11 studies concluded that the pooled effect size of change scores of depression scales was significant (SMD 0.25, 0.06‐0.44) only among studies concerned independent depression and was insignificant across all studies. A more recent meta‐analysis^102^ included 14 studies indicated that antidepressants were significantly better than placebo on reducing the endpoint scores of interviewer‐rated depression scales (SMD −0.27, −0.49 to −0.04). Our findings were consistent with the latter one and also indicated that non‐antidepressants revealed no benefit to depressive symptoms. Since antidepressants included various types and with quite different mechanisms and efficacy, thus, one of the innovations of our studies was to divide antidepressants into several subtypes, which was more meaningful for clinical practice. Besides, we included a wide spectrum of depressive disorders, including depressive symptoms measured by validated interviewer‐rated or self‐rated scales, and depression ascertained by standard diagnostic criteria, to fully represent the situation encountered by clinicians. According to our findings, noradrenaline reuptake inhibitor was hierarchically the best in reducing the depression scores. When the severity of depression was taken into account, in addition to NRI, another antidepressant‐mirtazapine that also targets at noradrenergic systems also revealed significantly better efficacy than controls in improving depressive symptoms. Notably, in direct pairwise analysis, the statistical significance of the result of NRI was contributed only to viloxazine (selective noradrenaline reuptake inhibitor) but not to venlafaxine (serotonin and noradrenaline reuptake inhibitor, SNRI). Since there was only one study regarding viloxazine included in our analysis, the network meta‐analysis result of NRI should be interpreted with caution and more future studies are warrant. In all, antidepressants, especially NRI and mirtazapine, might be the promising pharmacotherapy for depressive symptoms for comorbidity patients. However, both NRI and mirtazapine showed no significant efficacy on alcohol‐related symptoms compared to controls, which indicated combination with medication specifically target at AUD symptoms (eg, disulfiram) was needed.

Interestedly, although not approved for the treatment of alcohol use disorders and mono‐depression, antiepileptics showed exciting potential and prospects in treatment both alcohol‐related and depressive symptoms according to our findings. Antiepileptics included in this analysis, topiramate and tiagabine, could modulate GABAergic systems in the central amygdala, which was a region proved to be involved in the emotion regulation and alcohol intake.^103^, ^104^ Besides, these antiepileptics could enhance the inhibitory function of GABA, antagonize the excitatory of the glutamate system, and inhibits dopamine release, which further modulated the reward systems and addictive behaviors.^105^ However, two of the included studies regarded antiepileptics were open‐label controlled studies,^27^, ^28^ and the total number was scarce, so the results should be interpreted with caution and more evidence for validation is warranted.

Several limitations existed in our studies. Firstly, due to the heterogeneity of tolerability data across studies, no attempt was made to compare the tolerability of different therapeutic options in the current network meta‐analysis. However, since the higher‐ranked therapeutic options are approved medication for the treatment of AUD or depression, we considered the tolerability acceptable. Besides, there were other alternatives with relatively higher ranks could be chosen based on the patients’ condition. Secondly, the NMA results and the cumulative probabilities ranking may be influenced by the limited numbers of included studies in some intervention groups which might also contribute to the local inconsistency detected in the analysis. Although the direct pairwise analysis was conducted to validate the main results, cautions still be needed when interpreted the results and more evidence is warranted. Finally, our analysis focused only on pharmacotherapy but not on psychotherapy, which might partly explain the weak benefit of pharmacotherapy showed to depressive symptoms in the current analysis. However, since psychotherapy was not contradicted with drug therapy and safe, we assumed that it was always beneficial or not harmful when used adjunctively to pharmacotherapy.

## 5 CONCLUSION

Our Bayesian network meta‐analysis indicated that disulfiram was demonstrated to be associated with the best efficacy in achieving remission and increasing abstinent days for patients who comorbid alcohol use disorders (AUD) and depressive symptoms. Noradrenaline reuptake inhibitors (NRI) might hieratically be the best in efficacy for depressive symptoms. Besides, antiepileptics might be beneficial to both alcohol‐related and depressive symptoms. However, more evidence is warranted to corroborate the findings above.

## CONFLICTS OF INTEREST

The authors declare no conflict of interest.

## AUTHOR CONTRIBUTIONS

Jiande Li and Ying Peng involved in conceptualization; Jiande Li, Hongxuan Wang, and Ying Peng involved in data search and extraction; Jiande Li and Hongxuan Wang involved in data analysis and software; Jiande Li, Mei Li, Qingyu Shen, and Xiangpen Li interpreted the results; Jiande Li and Hongxuan Wang involved in software; Jiande Li wrote the original draft; Xiaoming Rong and Ying Peng wrote and edited the article.

## ETHICAL STATEMENT

The current study does not require an ethical statement.

## Supporting information

Supplementary Material
