# Supplementary material for: Efficacy of pharmacotherapeutics for patients comorbid with alcohol use disorders and depressive symptoms—A bayesian network meta‐analysis
Source: CNS Neurosci Ther. 2020 Jul 19;26(11):1185–97. doi: 10.1111/cns.13437 (PMC7564195; doi:10.1111/cns.13437)
Supplement: Supplementary file 1 — Supplementary Material [file CNS-26-1185-s001.docx]

**Supplementary table 1.** Search strategies used in PubMed.

| **#** | **Search strategies** | **Hits** |
| --- | --- | --- |
| **1** | Alcohol-Related Disorders[MeSH Terms] OR Drinking behaviour[MeSH Terms] OR alcoholism[MeSH Terms] OR AUD[Title/Abstract] OR drink*[Title/Abstract] OR alcoholic*[Title/Abstract] OR (alcohol*[Title/Abstract]) AND (dependen*[Title/Abstract] OR disorder*[Title/Abstract] OR misuse*[Title/Abstract] OR abuse*[Title/Abstract] OR consumption*[Title/Abstract]) | 249,666 |
| **2** | "depressive disorder"[MeSH Terms] OR "mood disorders"[MeSH Terms] OR "affective symptoms"[MeSH Terms] OR "affective disorders, Psychotic"[MeSH Terms] OR depress*[Title/Abstract] OR dysthymi*[Title/Abstract] OR "affective disorder*"[Title/Abstract] OR mdd[Title/Abstract] | 494,008 |
| **3** | (((((((("drug therapy"[Subheading] OR "drug therapy"[Mesh] OR "drug treatment"[Title/Abstract] OR medication[Title/Abstract] OR pharmacotherapy[Title/Abstract] OR pharmacological[Title/Abstract] OR medicine[Title/Abstract] OR "Pharmacological intervention*"[Title/Abstract] OR ((drug[Title/Abstract] OR drugs[Title/Abstract] OR pharmacologic*[Title/Abstract]) AND treatment*[Title/Abstract]))) OR (Disulfiram[MeSH Terms] OR Baclofen[MeSH Terms] OR Naltrexone[MeSH Terms] OR acamprosate[MeSH Terms] OR Topiramate[MeSH Terms] OR Disulfiram OR Baclofen OR Naltrexone OR acamprosate OR Nalmefene OR Topiramate)) OR (Antidepressive Agents[Mesh] OR Monoamine Oxidase Inhibitors[Mesh] OR Serotonin Uptake Inhibitors[Mesh] OR anti-depres*[Title/Abstract] OR antidepres*[Title/Abstract] OR "monoamine oxidase inhibitors" OR "selective serotonin reuptake inhibitors" OR "tricyclic Drugs" OR SSRI* OR SNRI* OR MAOI* OR acetylcarnitine OR agomelatine OR alaproclate OR amesergide OR amiflamine OR amineptine OR amitriptyline OR amoxapine OR agomelatine OR Atomoxetine OR aprepitant OR befloxatone OR benactyzine OR brofaromine OR bupropion OR butriptyline OR caroxazone OR chlorproxithene OR cilobamine OR cimoxatone OR citalopram OR clomipramine OR clorgyline OR chlorimipramine OR clovoxamine OR deanol OR demexiptiline OR deprenyl OR desipramine OR dibenzepin OR diclofensine OR dothiepin OR doxepin OR duloxetine OR escitalopram OR etoperidone OR femoxetine OR fluotracen OR fluoxetine OR fluparoxan OR fluvoxamine OR idazoxan OR imipramine OR Iprindol* OR iproniazid OR isocarboxazid OR ketamine OR litoxetin* OR lofepramin* OR maprotilin* OR Medifoxamin* OR melitracene OR metapramin* OR mianserin OR milnacipran OR minaprin* OR mirtazapin* OR moclobemid* OR nefazodon* OR nialamid* OR nomifensin* OR nortriptylin* OR noxiptilin* OR opipramol OR oxaflozan* OR oxaprotilin* OR pargylin* OR paroxetin* OR phenelzin* OR piribedil OR pirlindol* OR pivagabin* OR prosulprid* OR protriptylin* OR quinupramin* OR reboxetin* OR ritanserin OR rolipram OR selegiline OR sertralin* OR setiptilin* OR teniloxazine OR tetrindol* OR thiazesim OR thozalinon* OR tianeptin* OR toloxaton* OR tomoxetin* OR tranylcypromin* OR trazodon* OR trimipramin* OR tryptophan OR venlafaxin* OR viloxazin* OR viqualin* OR zimeldin* )) OR (Antipsychotic Agents[Mesh] OR Anti-psychotic*[Title/Abstract] OR Antipsychotic*[Title/Abstract] OR amisulpride OR aripiprazole OR benperidol OR benfotiamine OR chlorpromazine OR clozapine OR decanoate OR embonate OR flupentixol OR "flupentixol decanoate" OR "fluphenazine decanoate" OR haloperidol OR hydrochloride OR levomepromazine OR mesoridazine OR olanzapine OR paliperidone OR pericyazine OR perphenazine OR pimozide OR prochlorperazine OR "promazine hydrochloride" OR "pipotiazine palmitate" OR quetiapine OR risperidone OR risperidone OR tiapride OR thioridazine OR trifluoperazine OR zuclopenthixol OR zuclopenthixol OR "zuclopenthixol acetate" )) OR (Benzodiazepines [Mesh] OR Benzodiazepin* or nitrazepam or Flurazepam or Loprazolam or Lormetazepam or lorazepam or temazepam or diazepam or alprazolam or chlordiazepoxide hydrochloride or oxazepam or Buspirone hydrochloride or meprobamate or Zaleplon or Zolpidem Tartrate or Zopiclone or Chloral hydrate or clomethiazole or Promethazine hydrochloride or Melatonin)) OR (Anticonvulsants[MeSH] OR anticonvulsant* OR acetazolamide OR amobarbital OR bromide* OR carbamazepine OR chlormethiazole OR clorazepate OR depakote OR depakene OR depakine OR divalproex OR ethosuximide OR felbamate OR fosphenytoin OR gabapentin OR lamotrigine OR levetiracetam OR metaclazepam OR lidocaine OR "magnesium sulphate" OR mephobarbital OR lignocaine OR memantine OR methsuximide OR mysoline OR mizodin OR oxcarbazepine OR paraldehyde OR phenobarbital OR pentobarbital OR phenytoin OR primidone OR promazine OR sartan OR tetrabamate OR tiagabine OR topamax OR topiramate OR valproic OR valproate OR vigabatrin OR zonisamide OR zonegran)) OR (mood stabilizer[Title/Abstract] OR Lithium)) | 4,348,566 |
| **4** | ((randomized controlled trial OR controlled clinical trial[pt] OR randomized[Title/Abstract] OR placebo[Title/Abstract] OR drug therapy[Title/Abstract] OR randomly[Title/Abstract] OR trial[Title/Abstract] OR groups[Title/Abstract]) NOT (animals[MeSH Terms] NOT humans[MeSH Terms])) | 2,608,643 |
| **5** | #1 AND #2 AND #3 AND #4 | 1381 |

**Supplementary table 2**. Characteristics of included studies.

| Author | Year | Region | Age  (mean±sd) | Percentages of female | Intervention  (maxium dose) | Controls | Treatment  Session  (weeks) | Sample sizes | | Measurement for depression | Outcome^*^ |  |
| --- | --- | --- | --- | --- | --- | --- | --- | --- | --- | --- | --- | --- |
|  |  |  |  |  |  |  |  | Intervention | Controls |  |  |  |
| Addolorato | 2002 | Italy | 47.5±10.5 | NA | Baclofen  (90mg/d) | Placebo | 4 | 20 | 19 | ZUNG | 1,2,3 |  |
| Altamura | 1990 | Italy | 44.5±14.4 | 20% | Viloxazine  (400mg/d) | Placebo | 12 | 15 | 15 | DSM-Ⅲ | 3 |  |
| Altintoprak | 2008 | Turkey | 44.0±7.9 | 6.5% | Mirtazapine  (65mg/d) | Amitriptyline  (150mg/d) | 8 | 24 | 20 | DSM-Ⅳ | 3 |  |
| Adamson | 2015 | New Zealand | 43.6±9.1 | 59.4% | Naltrexone+ citalopram  (100 mg/d+200 mg/d) | Naltrexone  (100 mg/d) | 12 | 73 | 65 | DSM-Ⅳ | 2,3 |  |
| Baltieri | 2008 | Netherland | 44.3±8.4 | 0 | Topiramate  (300 mg/d)  Naltrexone  (50 mg/d) | Placebo | 12 | 52  49 | 54 | HAMD | 1,2 | |
| Beraha | 2016 | Netherland | 44.8±9.6 | 31.1% | Baclofen  (150 mg/d) | Placebo | 16 | 89 | 62 | BDI | 1,2,3 | |
| Besson | 1998 | France | 42.5±NA | 20% | Acamprosate  (2000 mg/d) | Placebo | 12 | 55 | 55 | HAMD | 1,2 | |
| Book | 2008 | USA | 29.0±7.5 | 47.6% | Paroxetine  (60 mg/d) | Placebo | 16 | 20 | 22 | BDI | 2,3 | |
| Batki | 2014 | USA | 50.0±13.3 | 6.7% | Topiramate  (300 mg/d) | Placebo | 12 | 14 | 16 | BDI-2 | 2,3 | |

* Outcome parameters included: 1). Alcohol use disorders remission rate; 2) percent abstinent days; 3) reduction in scores of depression scales.

(Continued)

| Author | Year | Region | Age  (mean±sd) | Percentages of female | Intervention  (maxium dose) | Controls | Treatment  Session  (weeks) | Sample sizes | | Measurement for depression | Outcome^*^ |
| --- | --- | --- | --- | --- | --- | --- | --- | --- | --- | --- | --- |
|  |  |  |  |  |  |  |  | Intervention | Controls |  |  |
| Brady | 2005 | USA | 36.7±8.5 | 49.0% | Sertraline  (150 mg/d) | Placebo | 12 | 49 | 45 | HAMD | 2 |
| Cocchi | 1997 | Italy | 42.2±10.6 | 43.3% | Paroxetine  (20 mg/d) | Amitriptyline  (25mg/d) | 4 | 61 | 61 | DSM-Ⅲ | 3 |
| Chick | 2004 | UK | 42±10 | 25% | Fluvoxamine  (300mg/d) | Placebo | 12 | 243 | 249 | HAMD | 1,2 |
| Ciraulo | 2013 | USA | NA | 22.2% | Venlafaxine  (225 mg/d) | Placebo | 12 | 38 | 43 | HAMD | 1,3 |
| Cornelius | 2016 | USA | 41.3±8.8 | 29% | Mirtazapine  (30 mg/d) | Placebo | 12 | 7 | 7 | DSM-Ⅳ | 2,3 |
| Cornelius | 1997 | USA | 35.5±10.1 | 44% | Fluoxetine  (40 mg/d) | Placebo | 12 | 12 | 13 | DSM-Ⅲ | 2 |
| Cornelius | 1997 | USA | 34.8±10.2 | 49% | Fluoxetine  (40 mg/d) | Placebo | 12 | 25 | 26 | DSM-Ⅲ | 1,2,3 |
| Dongier | 1991 | Canada | 42.8±9.6 | 23.7% | Bromocriptine  (7.5 mg/d) | Placebo | 8 | 18 | 20 | HAMD | 1,2,3 |
| Dorus | 1989 | USA | 41.1±9.1 | 0% | Lithium  (1200 mg/d) | Placebo | 52 | 76 | 88 | DSM-Ⅲ | 1,2,3 |
| Fawcett | 1987 | USA | 39.3±9.9 | 16.3% | Lithium  (900 mg/d) | Placebo | 52 | 33 | 32 | HAMD | 1,3 |
| Fawcett | 2000 | USA | 40.5±7 | 0% | Buspirone  (40 mg/d)  Lithium  (1200 mg) | Placebo | 28 | 48  56 | 52 | BDI | 2 |

* Outcome parameters included: 1). Alcohol use disorders remission rate; 2) percent abstinent days; 3) reduction in scores of depression scales.

| Author | Year | Region | Age  (mean±sd) | Percentages of female | Intervention  (maxium dose) | Controls | Treatment  Session  (weeks) | Sample sizes | | Measurement for depression | Outcome^*^ |
| --- | --- | --- | --- | --- | --- | --- | --- | --- | --- | --- | --- |
|  |  |  |  |  |  |  |  | Intervention | Controls |  |  |
| Goyer | 1984 | USA | 33.2±9.8 | 0% | Disulfiram  (500 mg) | Placebo | 3 | 24 | 12 | ZUNG | 3 |
| Gual | 2003 | Spain | 46.7±9.5 | 47% | Sertraline  (150 mg) | Placebo | 24 | 44 | 39 | DSM-Ⅳ | 1,2,3 |
| Guardia | 2004 | Spain | 43.4±12.1 | 23.3% | Olanzapine  (15 mg) | Placebo | 12 | 29 | 31 | BDI | 1,2,3 |
| Gupta | 2017 | India | 36.3±9.5 | NA | Baclofen  (30 mg/d) | Placebo | 12 | 72 | 50 | HAMD | 1,2,3 |
| Habrat | 2006 | Poland | NA | NA | Tianeptine  (37.5 mg/d) | fluvoxamine (100 mg/day) | 6 | 150 | 148 | HAMD | 3 |
| Han | 2013 | Korea | 39.6±8 | 22.9% | Aripiprazole  (15 mg/d) | Placebo | 6 | 17 | 18 | DSM-Ⅳ | 1,3 |
| Hauser | 2017 | USA | 57±NA | 1.7% | Baclofen  (30 mg/d) | Placebo | 12 | 79 | 89 | BDI-2 | 1,2,3 |
| Hernandez-Avila | 2004 | USA | 42.9±8.6 | 51% | Nefazodone  (600 mg/d) | Placebo | 10 | 21 | 20 | DSM-Ⅳ | 1,2,3 |
| Janiri | 1996 | Italy | 45.4±17.6 | 20% | Fluoxetine  20mg/d | Placebo | 8 | 21 | 29 | HAMD | 1,3 |
| Kranzler | 1994 | USA | 39.4±9.3 | 23% | Buspirone  60mg/d | Placebo | 12 | 31 | 27 | HAMD | 1,2 |
| Kranzler | 2006 | USA | 42.7±8.9 | 36.2% | Sertraline  (200 mg/d) | Placebo | 10 | 159 | 169 | DSM-Ⅳ | 2,3 |

* Outcome parameters included: 1). Alcohol use disorders remission rate; 2) percent abstinent days; 3) reduction in scores of depression scales.

(Continued)

| Author | Year | Region | Age  (mean±sd) | Percentages of female | Intervention  (maxium dose) | Controls | Treatment  Session  (weeks) | Sample sizes | | Measurement for depression | Outcome^*^ |
| --- | --- | --- | --- | --- | --- | --- | --- | --- | --- | --- | --- |
|  |  |  |  |  |  |  |  | Intervention | Controls |  |  |
| Krupitsky | 1993 | Russia | 37.1±7.4 | NA | Baclofen  (37.5 mg/d)  Amitriptyline  (75 mg/d) | Placebo | 3 | 29  18 | 23 | ZUNG | 3 |
| Laaksonen | 2008 | Finland | 43.1±8.6 | 29.2% | Naltrexone  (50 mg/d)  Disulfiram  (200 mg/d) | Acamprosate  (1998 mg/d) | 12 | 50  39 | 50 | BDI | 1,2 |
| Latt | 2002 | Australia | 44.8±10.6 | 30.8% | Naltrexone  (50 mg/d) | Placebo | 12 | 56 | 51 | BDI | 1,2,3 |
| Le Bon | 2003 | Belgium | 43.8±8.3 | 5.6% | Trazodone  (200 mg/d) | Placebo | 4 | 8 | 8 | HAMD | 1,3 |
| Lippas | 2004 | Greece | 44.6±9.4 | 19.5% | Mirtazapine  (60 mg/d) | Placebo | 4 | 39 | 38 | HAMD | 3 |
| Lippas | 2005 | Greece | 47.2±10.6 | 28.3% | Mirtazapine  (60 mg/d)  Venlafaxine  (300 mg/d) | Placebo | 5 | 20  20 | 20 | HAMD | 3 |
| Malec | 1996 | Canada | 41.6±7.7 | 17.5% | Buspirone  (40 mg/d) | Placebo | 12 | 28 | 29 | MARDS | 1,2 |
| Mason | 1996 | USA | 39.5±16.2 | 17% | Desipramine  (200 mg/d) | Placebo | 26 | 12 | 10 | DSM-Ⅲ | 1 |

* Outcome parameters included: 1). Alcohol use disorders remission rate; 2) percent abstinent days; 3) reduction in scores of depression scales.

(Continued)

| Author | Year | Region | Age  (mean±sd) | Percentages of female | Intervention  (maxium dose) | Controls | Treatment  Session  (weeks) | Sample sizes | | Measurement for depression | Outcome^*^ |
| --- | --- | --- | --- | --- | --- | --- | --- | --- | --- | --- | --- |
|  |  |  |  |  |  |  |  | Intervention | Controls |  |  |
| McGrath | 1996 | USA | 38.9±8.0 | 50.8% | Imipramine  (300 mg/d) | Placebo | 12 | 27 | 29 | DSM-Ⅲ | 1,2,3 |
| Merry | 1976 | UK | 42.9±13.5 | 43.8% | Lithium  (1200 mg/d) | Placebo | 6 | 9 | 7 | BDI | 1,2 |
| Moak | 2003 | USA | 41.5±10.4 | 39% | Sertraline  (200 mg/d) | Placebo | 12 | 38 | 44 | DSM-Ⅲ | 1,2,3 |
| Morley | 2018 | Australia | 48±10 | 29% | Baclofen  (75 mg/d) | Placebo | 12 | 71 | 33 | DASS | 1,2,3 |
| Morley | 2006 | Australia | 45±9 | 30% | Acamprosate  (1998 mg/d)  Naltrexone  (50 mg/d) | Placebo | 12 | 55  53 | 61 | DASS | 1,2 |
| Morgenstern | 2012 | USA | 40.4±11.5 | 0 | Naltrexone  (100 mg/d) | Placebo | 12 | 93 | 93 | BDI-2 | 1,2,3 |
| Mueller | 1997 | USA | 38.6±8.5 | 37.9% | Carbamazepine  (600 mg/d) | Placebo | 16 | 12 | 15 | BDI | 1 |
| Muhonen | 2008 | Finland | 47.7±8.3 | 55% | Memantine  (20 mg/d) | Citalopram  20mg/d | 26 | 40 | 40 | DSM-Ⅳ | 1 |
| Nunes | 1993 | USA | 40±9 | 53% | Imipramine  (300 mg/d) | Placebo | 26 | 13 | 10 | DSM-Ⅲ | 1 |
| O’Marley | 2008 | 美国 | 40±9.8 | 34% | Naltrexone  (50 mg/d)  Naltrexone+Sertraline  (50 mg/d+100 mg/d) | Placebo | 16 | 34  33 | 34 | CESD | 1,2,3 |

* Outcome parameters included: 1). Alcohol use disorders remission rate; 2) percent abstinent days; 3) reduction in scores of depression scales.

(Continued)

| Author | Year | Region | Age  (mean±sd) | Percentages of female | Intervention  (maxium dose) | Controls | Treatment  Session  (weeks) | Sample sizes | | Measurement for depression | Outcome^*^ |
| --- | --- | --- | --- | --- | --- | --- | --- | --- | --- | --- | --- |
|  |  |  |  |  |  |  |  | Intervention | Controls |  |  |
| Oslin | 2005 | USA | 63.4±6.3 | 20.3% | Naltrexone  (50 mg/d) | Placebo | 12 | 37 | 37 | DSM-Ⅳ | 1 |
| Paparrigopoulos | 2011 | Greece | 45.4±10.1 | 11.8% | Topiramate  (75 mg/d) | Placebo | 16 | 30 | 55 | HAMD | 1,3 |
| Paparrigopoulos | 2010 | Greece | 46.8±7.7 | 25.8% | Tiagabine  (20 mg/d) | Placebo | 26 | 57 | 56 | HAMD | 1,3 |
| Petrakis | 2007 | USA | 47.1±8.6 | 3.6% | Naltrexone  (50 mg/d)  Disulfiram  (250 mg)  Naltrexone+ Disulfiram  (50 mg/d+250 mg/d) | Placebo | 12 | 34  43  28 | 34 | DSM-Ⅳ | 1,2,3 |
| Petrakis | 2012 | USA | 47.1±8.9 | 9.1% | Naltrexone+  Paroxetine  (50 mg/d+40 mg/d)  Desipramine  (200 mg/d) | Paroxetine  (40 mg/d) | 10 | 22  24 | 20 | HAMD | 3 |
| Pettinati | 2000 | USA | 40.6±8.5 | 42.2% | Sertraline  (200 mg/d) | Placebo | 14 | 20 | 25 | HAMD | 1,2 |
| Pettinati | 2001 | USA | 44.6±10.2 | 48% | Sertraline  (200 mg/d) | Placebo | 14 | 26 | 27 | DSM-III | 1,2,3 |
| Poldrugo | 1997 | Italy | 43.9±9.7 | 72.8% | Acamprosate  1998mg/d | Placebo | 26 | 122 | 124 | HAMD | 1,2 |

* Outcome parameters included: 1). Alcohol use disorders remission rate; 2) percent abstinent days; 3) reduction in scores of depression scales.

(Continued)

| Author | Year | Region | Age  (mean±sd) | Percentages of female | Intervention  (maxium dose) | Controls | Treatment  Session  (weeks) | Sample sizes | | Measurement for depression | Outcome^*^ |
| --- | --- | --- | --- | --- | --- | --- | --- | --- | --- | --- | --- |
|  |  |  |  |  |  |  |  | Intervention | Controls |  |  |
| Pettinati | 2010 | USA | 43.3±9.6 | 37.6% | Naltrexone  (100 mg/d)  Sertraline  (200 mg/d)  Naltrexone+Sertraline | Placebo | 14 | 49  40  42 | 39 | HAMD | 1,3 |
| Ponizovsky | 2015 | Israel | 43.7±9.1 | 25% | Baclofen  (50 mg/d) | Placebo | 12 | 32 | 32 | BDI | 2,3 |
| Roy | 1998 | USA | 40.9±6.6 | 8.3% | Sertraline  (100 mg/d) | Placebo | 8 | 18 | 18 | DSM-III | 3 |
| Roy-Byrne | 2000 | USA | 39.5±7.5 | 62.5% | Nefazodone  (500 mg/d) | Placebo | 12 | 31 | 25 | DSM-III | 1,3 |
| Shaw | 1975 | UK | 31.6±NA | 0 | Imipramine  (150 mg/d) | Placebo | 5 | 29 | 29 | ZUNG | 3 |
| Sonne | 2006 | USA | 44.1±9.3 | 38.9% | Acamprosate  1998mg/d | Placebo | 12 | 45 | 45 | DSM-Ⅳ | 2,3 |
| Stella | 2008 | Italy | 41.8±12 | 29.2% | Naltrexone+Citalopram  (75 mg/d+20mg/d) | Citalopram  (20 mg/d) | 26 | 23 | 24 | HAMD | 1,3 |
| Tiihonen | 1992 | USA | 45.8±7.9 | 0 | Citalopram  (40 mg/d) | Placebo | 12 | 31 | 31 | ZUNG | 3 |
| Tollefson | 1992 | USA | 38.4±0.4 | 27.5% | Buspirone  (60 mg/d) | Placebo | 24 | 21 | 21 | HAMD  (15) | 3 |
| Witte | 2012 | USA | 46±14 | 46% | Acamprosate  (2000 mg/d) | Placebo | 12 | 12 | 11 | DSM-Ⅳ | 2,3 |
| NCT02646449 | 2016 | USA | 22.9±2.8 | 42.9% | Mirtazapine  (30 mg/d) | Placebo | 12 | 4 | 4 | DSM-Ⅳ | 3 |


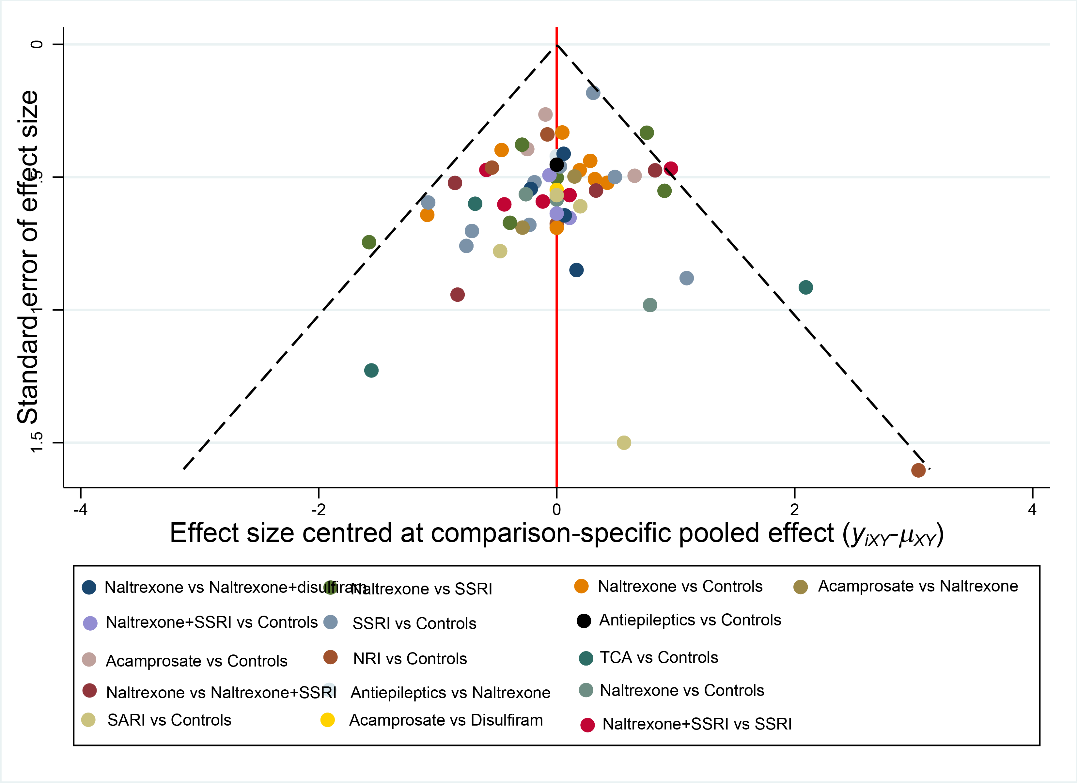


**Supplementary Figure 1A.** Publication bias of studies on AUD remission rate was assessed by funnel plots. All studies are centered on the summary effect estimate of their respective comparisons [μXY (logOR for the present study)], which is represented by the vertical red line.


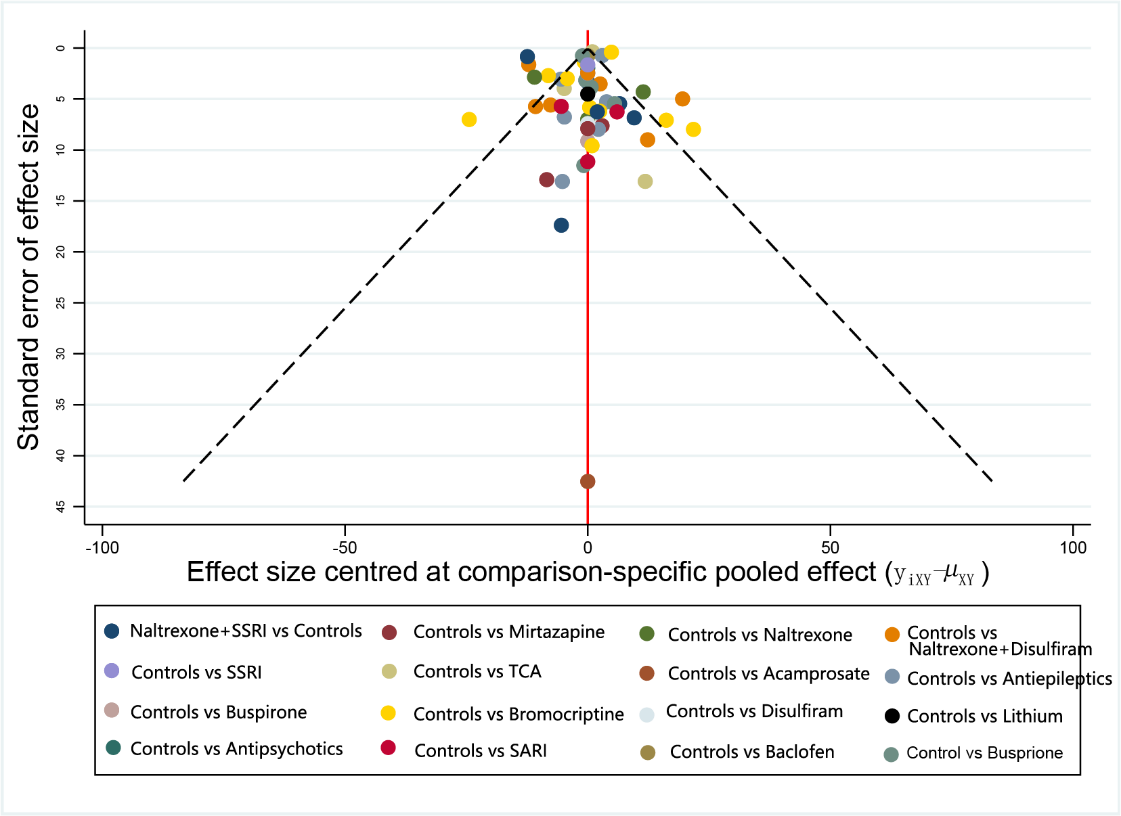


**Supplementary Figure 1B.** Publication bias of studies on percent abstinent days was assessed by funnel plots. All studies are centered on the summary effect estimate of their respective comparisons [μXY (logOR for the present study)], which is represented by the vertical red line.


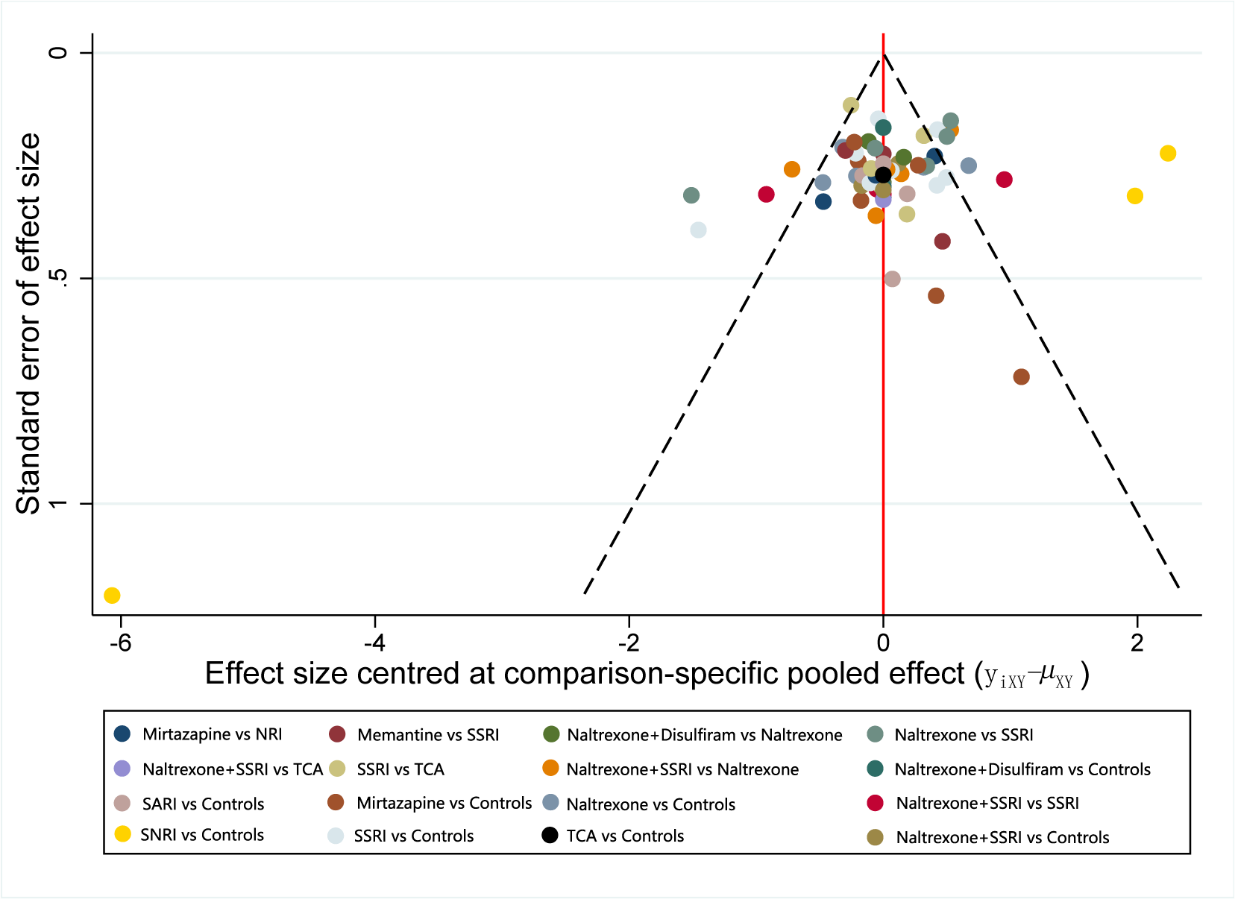


**Supplementary Figure 1C.** Publication bias of studies on percent abstinent days was assessed by funnel plots. All studies are centered on the summary effect estimate of their respective comparisons [μXY (logOR for the present study)], which is represented by the vertical red line.


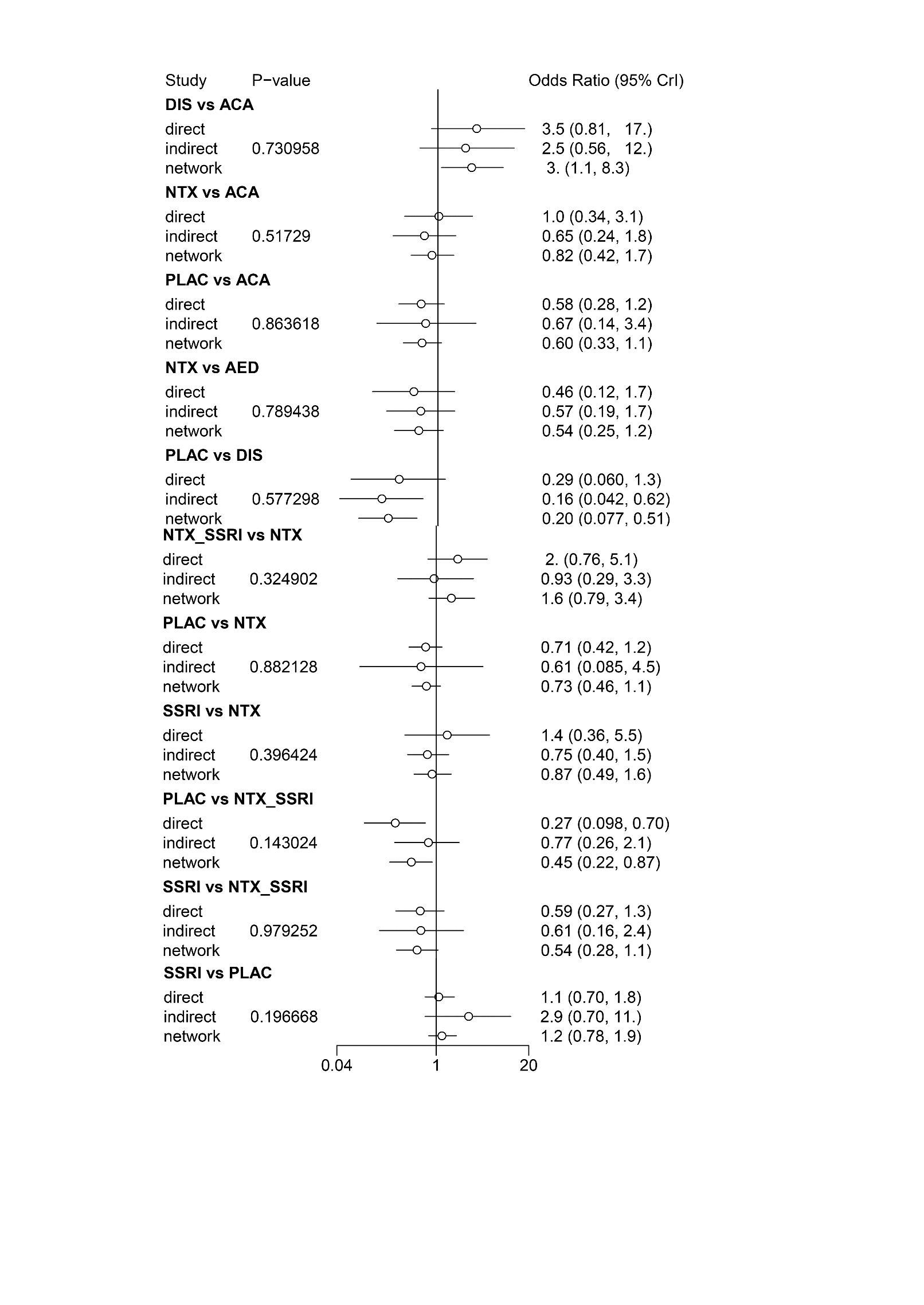


**Supplementary Figure 2A.** Results of node-splitting analysis. P value >0.05 represents that there is no significant difference between the direct and indirect results of the comparison.

ACA, acamprosate; AED, antiepileptics; DIS, disulfiram; NTX, naltrexone; SSRI, selective serotonin reuptake inhibitor.


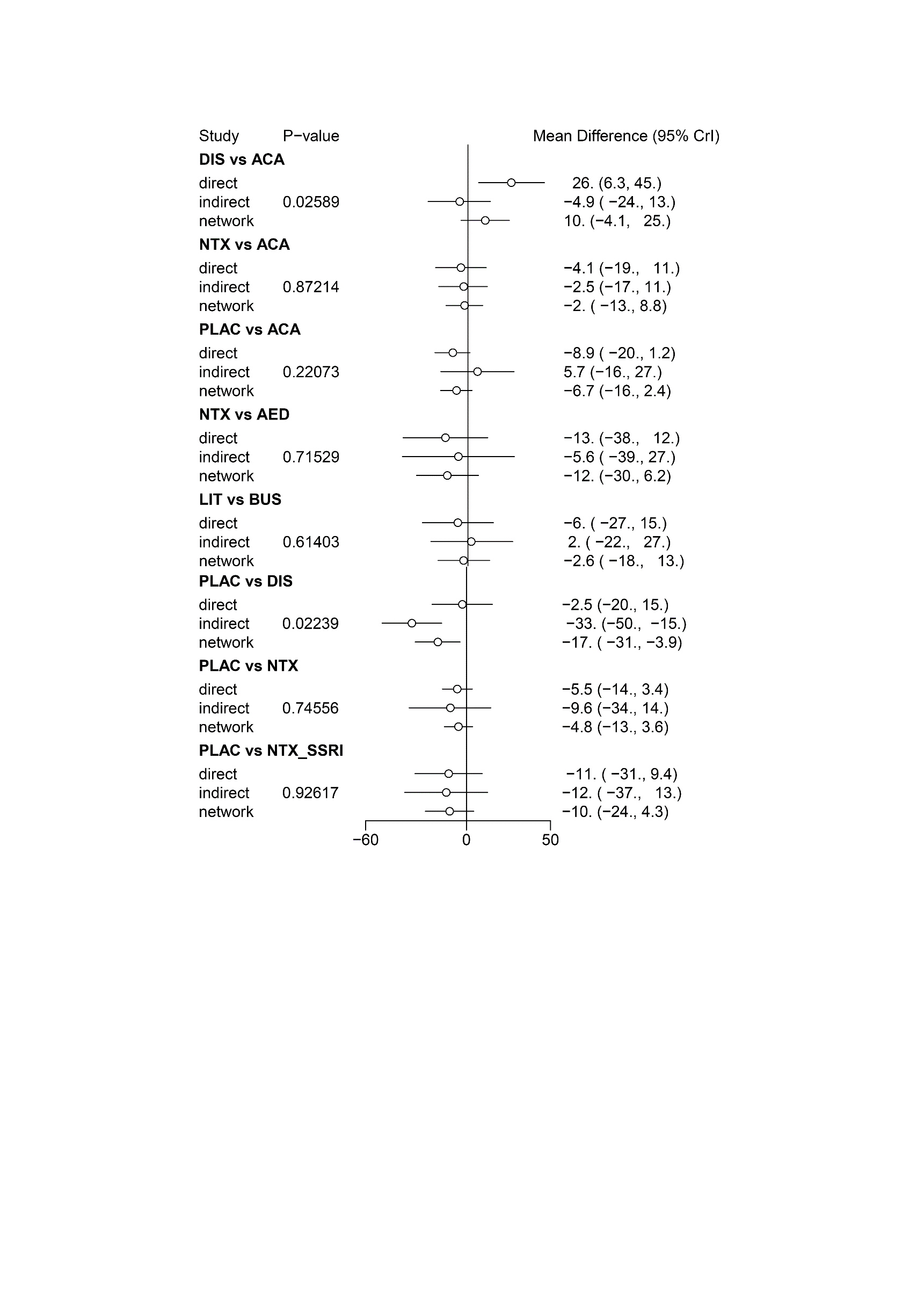


**Supplementary Figure 2B.** Results of node-splitting analysis. P value >0.05 represents that there is no significant difference between the direct and indirect results of the comparison.

ACA, acamprosate; AED, antiepileptics; BUS, buspirone; DIS, disulfiram; NTX, naltrexone; SSRI, selective serotonin reuptake inhibitor.


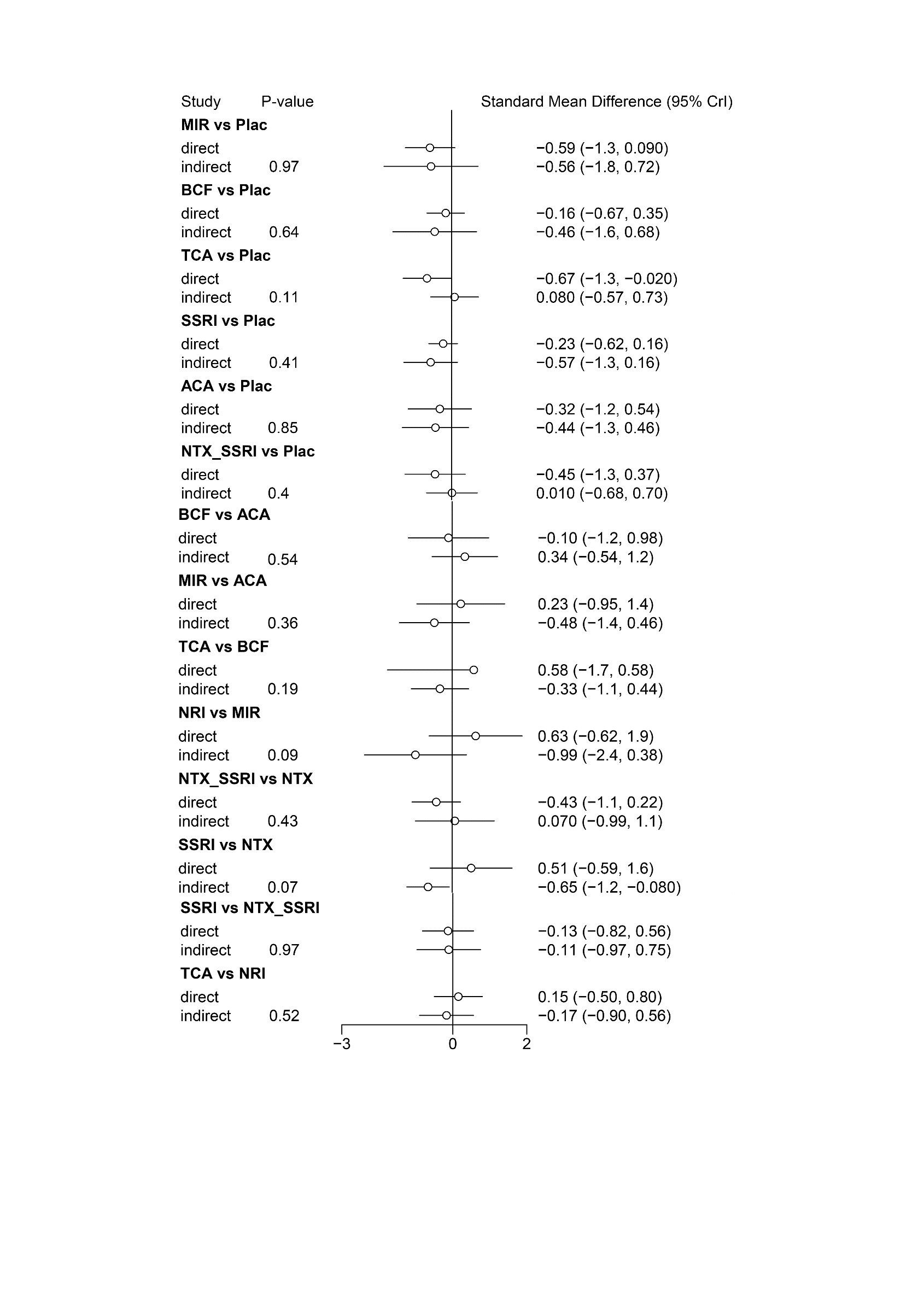


**Supplementary Figure 2C.** Results of node-splitting analysis. P value >0.05 represents that there is no significant difference between the direct and indirect results of the comparison.

ACA, acamprosate; BCF, baclofen; MIR, mirtazapine; NTX, naltrexone; NRI, noradrenaline reuptake inhibitor; SSRI, selective serotonin reuptake inhibitor; TCA, tricyclic antidepressants

**Supplementary table 3**. Main analysis and sensitivity analysis that included only participants with at least moderate depressive symptoms or with diagnosis of depression for three outcome parameters.

| Intervention | Main analysis | | | Studies with participants with at least moderate depressive symptoms or with diagnosis of depression | | |
| --- | --- | --- | --- | --- | --- | --- |
|  | AR | PAD | DEP | AR | PAD | DEP |
|  | Odds ratio | Mean difference | Std. mean difference | Odds ratio | Mean difference | Std. mean difference |
| Acamprosate | 1.66 (0.89, 3.05) | 6.79 (−2.30, 16.19) | −0.42 (−1.45, 0.60) | 1.71 (0.84, 3.41) | 9.45 (−0.56, 19.95) | −0.62 (−1.67, 0.42) |
| Antiepileptics | **2.55 (1.26, 5.22)** | 16.94 (−0.80, 34.58) | −0.70 (−2.05, 0.65) | 2.54 (0.98, 6.67) | 10.06 (−21.57, 41.85) | −0.70 (−2.04, 0.64) |
| Antipsychotics | 0.97 (0.30, 3.22) | 9.56 ( −13.88, 32.99) | −0.03 (−1.42, 1.36) | 0.97 (0.30, 3.3) | 9.58 (−14.15, 33.29) | −0.03 (−1.42, 1.36) |
| Baclofen | 1.75 (0.97, 3.29) | **10.42 (1.70, 19.33)** | −0.25 (−1.03, 0.54) | 1.27 (0.51, 3.47) | 9.50 (−7.26, 26.86) | −0.81 (−1.93, 0.30) |
| Buspirone | 1.70 (0.50, 5.65) | 7.51 (−4.83, 19.91) | −0.48 (−2.44, 1.48) | 2.38 (0.53, 10.8) | 7.08 (−14.15, 27.98) | NA |
| Bromocriptine | 0.37 (0.07, 1.89) | 4.29 (−24.62, 33.18) | −0.14 (−2.11, 1.83) | 0.36 (0.06, 1.94) | 4.21 (−24.92, 33.37) | −0.14 (−2.10, 1.83) |
| Disulfiram | **5.00 (1.97, 12.95)** | **17.03 (3.83, 30.47)** | 0.31 (−0.99, 1.61) | **4.34 (1.14, 17.51)** | 4.31 (−13.86, 27.98) | 0.28 (−1.01, 1.58) |
| Lithium | 0.70 (0.29, 1.52) | 4.98 (−7.23, 17.41) | 0.01 (−1.35, 1.37) | 0.70 (0.27, 1.5) | 8.46 (−7.71, 25.43) | −0.24 (−2.14, 1.66) |
| Memantine | 1.10 (0.29, 4.60) | NA | −0.50 (−2.51, 1.50) | 1.40 (0.32, 6.11) | NA | −0.52 (−2.53, 1.49) |
| Mirtazapine | NA | −0.95 ( −86.7, 83.8) | −0.78 (−1.69, 0.13) | NA | −0.56 (−85.58, 83.88) | **−0.99 (−1.91, -0.07)** |
| Naltrexone | 1.38 (0.88, 2.18) | 4.76 (−3.59, 13.13) | 0.11 (−0.68, 0.89) | 1.50 (0.89, 2.64) | 4.34 (−5.36, 13.90) | 0.02 (−0.85, 0.90) |
| Naltrexone plus disulfiram | 2.60 (0.71, 10.15) | 10.72 (−6.11, 27.65) | 0.30 (−1.37, 1.98) | 2.57 (0.66, 10.61) | 6.21 ( −11.69, 24.10) | 0.26 (−1.42, 1.94) |
| Naltrexone plus SSRI | **2.24 (1.15, 4.50)** | 10.01 (−4.32, 24.42) | −0.19 (−1.07, 0.68) | **2.56 (1.24, 5.37)** | 9.70 (−5.30, 24.75) | −0.23 (−1.22, 0.76) |
| SARI | 1.85 (0.62, 5.66) | 19.49 (−6.59, 45.62) | −0.23 (−1.38, 0.92) | 1.99 (0.61, 6.68) | 19.48 (−6.89, 45.83) | −0.25 (−1.62, 1.13) |
| NRI | 1.15 (0.21, 6.13) | NA | **−2.44 (−3.53, −1.36)** | NA | NA | **−3.60 (−4.91, −2.29)** |
| SSRI | 1.21 (0.78, 1.92) | 0.45 (−6.35, 7.36) | −0.33 (−0.90, 0.24) | 1.49 (0.86, 2.62) | 2.67 (−5.62, 11.06) | −0.35 (−0.95, 0.26) |
| TCA | 1.65 (0.57, 4.73) | 2.50 (−21.44, 26.40) | −0.31 (−1.11, 0.49) | 1.65 (0.56, 4.86) | 2.53 ( −21.71, 26.77) | −0.41 (−1.29, 0.47) |

AR, Alcohol use disorders remission rate; PAD, percent abstinent days; DEP, reduction in scores of depression scales.

**Supplementary table 4**. Main analysis and sensitivity analysis that included only studies with treatment sessions ≥8 weeks for three outcome parameters.

| Intervention | Main analysis | | | Studies with treatment sessions ≥8 weeks | | |
| --- | --- | --- | --- | --- | --- | --- |
|  | AR | PAD | DEP | AR | PAD | DEP |
|  | Odds ratio | Mean difference | Std. mean difference | Odds ratio | Mean difference | Std. mean difference |
| Acamprosate | 1.66 (0.89, 3.05) | 6.79 (−2.30, 16.19) | −0.42 (−1.45, 0.60) | 1.68 (0.97, 2.89) | 6.83 (−2.52, 16.43) | −0.18 (−1.25, 0.89) |
| Antiepileptics | **2.55 (1.26, 5.22)** | 16.94 (−0.80, 34.58) | −0.70 (−2.05, 0.65) | **2.55 (1.34, 4.89)** | 16.93 (−1.13, 34.89) | −0.70 (−2.04, 0.63) |
| Antipsychotics | 0.97 (0.30, 3.22) | 9.56 ( −13.88, 32.99) | −0.03 (−1.42, 1.36) | 0.66 (0.17, 2.48) | 9.56 (−14.34, 33.47) | −0.01 (−1.90, 1.93) |
| Baclofen | 1.75 (0.97, 3.29) | **10.42 (1.70, 19.33)** | −0.25 (−1.03, 0.54) | 1.37 (0.77, 2.45) | 9.78 (−0.09, 19.95) | −0.07 (−0.81, 0.94) |
| Buspirone | 1.70 (0.50, 5.65) | 7.51 (−4.83, 19.91) | −0.48 (−2.44, 1.48) | 1.72 (0.55, 5.30) | 7.04 (−5.74, 19.77) | −0.48 (−2.42, 1.46) |
| Bromocriptine | 0.37 (0.07, 1.89) | 4.29 (−24.62, 33.18) | −0.14 (−2.11, 1.83) | 0.37 (0.07, 1.71) | 4.25 (−25.04, 34.55) | −0.14 (−2.09, 1.82) |
| Disulfiram | **5.00 (1.97, 12.95)** | **17.03 (3.83, 30.47)** | 0.31 (−0.99, 1.61) | **4.97 (2.13, 11.94)** | **17.08 (3.48, 30.93)** | 0.22 (−1.48, 1.92) |
| Lithium | 0.70 (0.29, 1.52) | 4.98 (−7.23, 17.41) | 0.01 (−1.35, 1.37) | 0.98 (0.46, 2.13) | 3.00 ( −10.9, 16.8) | 0.01 (−1.34, 1.36) |
| Memantine | 1.10 (0.29, 4.60) | NA | −0.50 (−2.51, 1.50) | 1.10 (0.33, 3.90) | NA | −0.46 (−2.45, 1.53) |
| Mirtazapine | NA | −0.95 ( −86.7, 83.8) | −0.78 (−1.69, 0.13) | NA | −0.39 ( −86.1, 84.5) | −0.11 (−1.65, 1.42) |
| Naltrexone | 1.38 (0.88, 2.18) | 4.76 (−3.59, 13.13) | 0.11 (−0.68, 0.89) | 1.37 (0.92, 2.07) | 4.77 (−3.83, 13.35) | 0.11 (−0.68, 0.89) |
| Naltrexone plus disulfiram | 2.60 (0.71, 10.15) | 10.72 (−6.11, 27.65) | 0.30 (−1.37, 1.98) | 2.59 (0.78, 9.32) | 10.74 (−6.66, 28.17) | 0.27 (−1.44, 1.99) |
| Naltrexone plus SSRI | **2.24 (1.15, 4.50)** | 10.01 (−4.32, 24.42) | −0.19 (−1.07, 0.68) | **2.21 (1.20, 4.14)** | 10. 03 (−4.73, 24.84) | −0.17 (−1.05, 0.71) |
| SARI | 1.85 (0.62, 5.66) | 19.49 (−6.59, 45.62) | −0.23 (−1.38, 0.92) | 1.98 (0.67, 6.02) | 19.54 (−7.03, 45.97) | −0.25 (−1.62, 1.12) |
| NRI | 1.15 (0.21, 6.13) | NA | **−2.44 (−3.53, −1.36)** | 1.15 (0.23, 5.55) | NA | **−3.96 (−5.33, −2.59)** |
| SSRI | 1.21 (0.78, 1.92) | 0.45 (−6.35, 7.36) | −0.33 (−0.90, 0.24) | 1.17 (0.80, 1.80) | 0.48 (−6.50, 7.56) | −0.28 (−0.87, 0.30) |
| TCA | 1.65 (0.57, 4.73) | 2.50 (−21.44, 26.40) | −0.31 (−1.11, 0.49) | 1.67 (0.62, 4.48) | 2.46 ( −21.99, 26.86) | −0.28 (−1.39, 0.83) |

AR, Alcohol use disorders remission rate; PAD, percent abstinent days; DEP, reduction in scores of depression scales.

| Intervention | Number of data sets | Pairwise results*  Odds ratio | I^2^ (%) | SUCRA (%) |
| --- | --- | --- | --- | --- |
| Disulfiram | 5 | **3.22 (1.67, 6.20)** | 0 | 95.1 |
| Antiepileptics | 5 | **2.32 (1.40, 3.84)** | 0 | 77.9 |
| Naltrexone+Disulfiram | 2 | 2.00 (0.71, 5.61) | 0 | 73.3 |
| Naltrexone+SSRI | 7 | **1.93 (1.06, 3.52)** | 41.3 | 72.6 |
| SARI | 3 | 1.76 (0.72, 4.32) | 0 | 60.4 |

**Supplementary table 5A.** Direct pairwise analysis on comparison between the top five intervention and their corresponding lower ranked interventions in efficacy of AUD remission rate.

* versus lower ranked intervention or control.

**Supplementary table 5B.** Direct pairwise analysis on comparison between the top five intervention and their corresponding lower ranked interventions in efficacy of percent abstinent days.

| Intervention | Number of data sets | Pairwise results*  Mean difference | I^2^ (%) | SUCRA (%) |
| --- | --- | --- | --- | --- |
| Disulfiram | 5 | **9.81 (0.34, 19.27)** | 93.2 | 79.6 |
| SARI | 1 | **19.50 (1.61, 37.39)** | **-** | 76.6 |
| Antiepileptics | 3 | **16.01 (4.40, 27.61)** | 0 | 76.1 |
| Baclofen | 6 | **10.76 (0.10, 21.42)** | 90.1 | 61.0 |
| Naltrexone+Disulfiram | 2 | **4.16 (1.08, 7.24)** | 0 | 59.4 |

* versus lower ranked intervention or control.

**Supplementary table 5C.** Direct pairwise analysis on comparison between the top five intervention and their corresponding lower ranked interventions in efficacy of reduction in scores of depression scales.

| Intervention | Number of datasets | Pairwise results*  Std. mean difference | I^2^ (%) | SUCRA (%) |
| --- | --- | --- | --- | --- |
| NRI | 4 | -1.39 (-3.15, 0.38) | 94.8 | 99.0 |
| Viloxazine | 1 | **-8.54 (-10.89, -6.19)** | - |  |
| Venlafaxine | 3 | 0.23 (-0.42,0.88) | 66.0 |  |
| Mirtazapine | 6 | -0.46 (-0.98, 0.03) | 59.6 | 73.3 |
| Antiepileptics | 2 | **-0.73 (-1.02, -0.46)** | 0 | 66.6 |
| Acamprosate | 4 | -0.19 (-0.60, 0.22) | 62.8 | 57.2 |
| Memantine | 1 | -0.18 (0.62, 0.26) | - | 56.8 |

* versus lower ranked intervention or control.


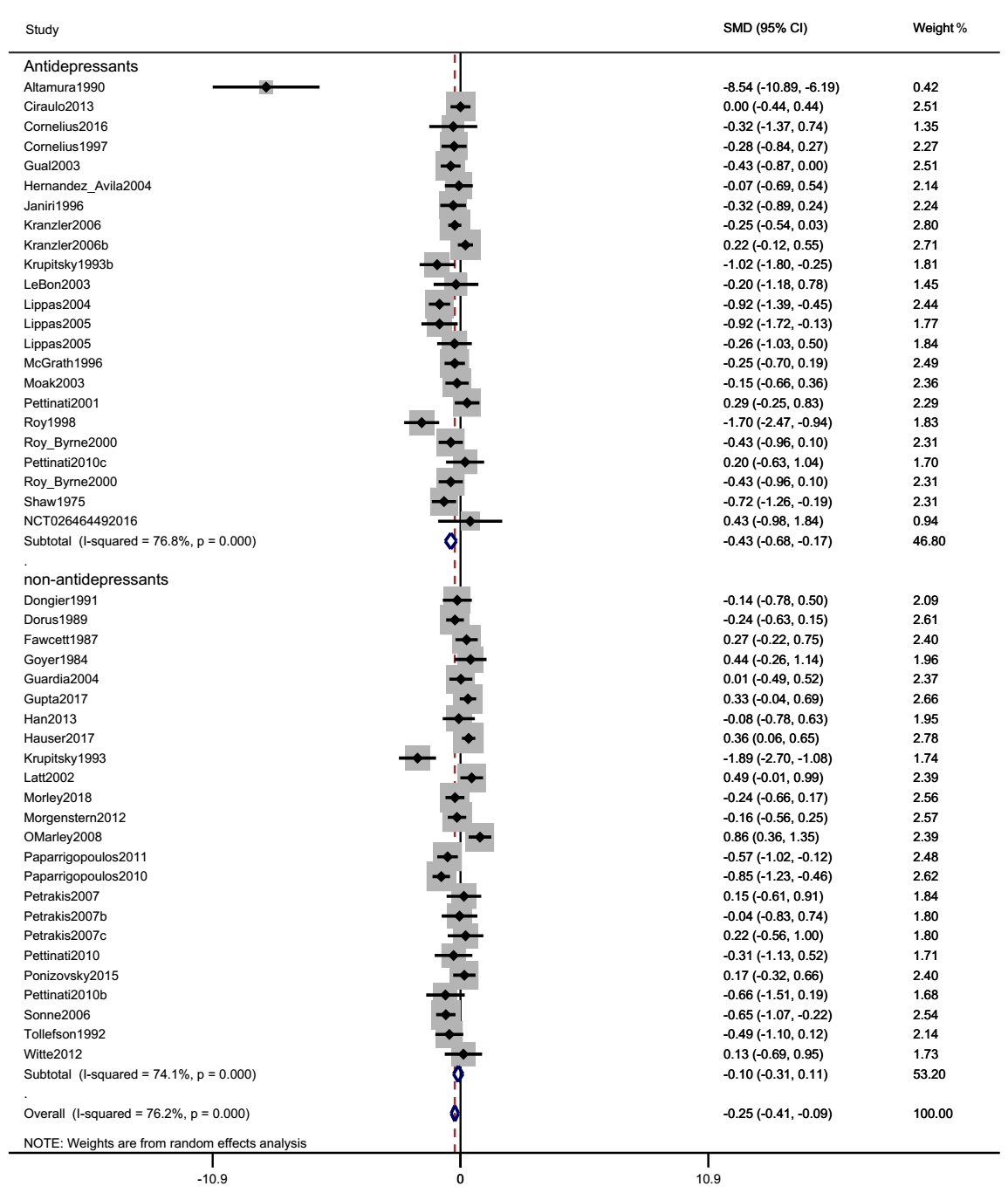
**Supplementary Figure 3A.** Forest plot of direct meta-analysis and subgroup analysis for efficacy of pharmacotherapy and antidepressants in reducing the depression scores.


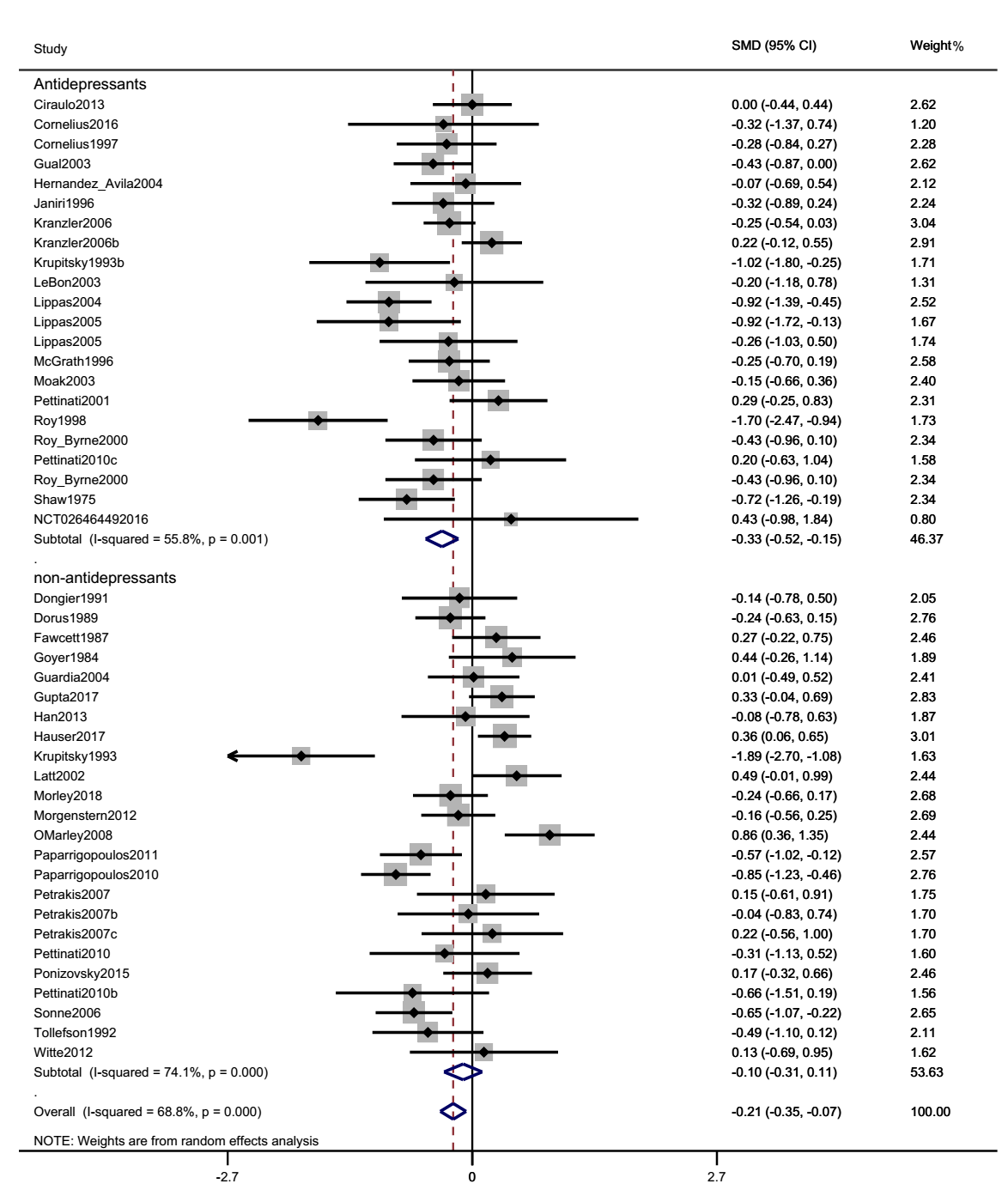


**Supplementary Figure 3B.** Forest plot of direct meta-analysis and subgroup analysis for efficacy of pharmacotherapy and antidepressants in reducing the depression scores after excluding one study (Altamura1990) contributing to heterogeneity.

**Supplementary Figure 4A.** Risk of bias for each included study using Cochrane’s risk of bias assessment tool.


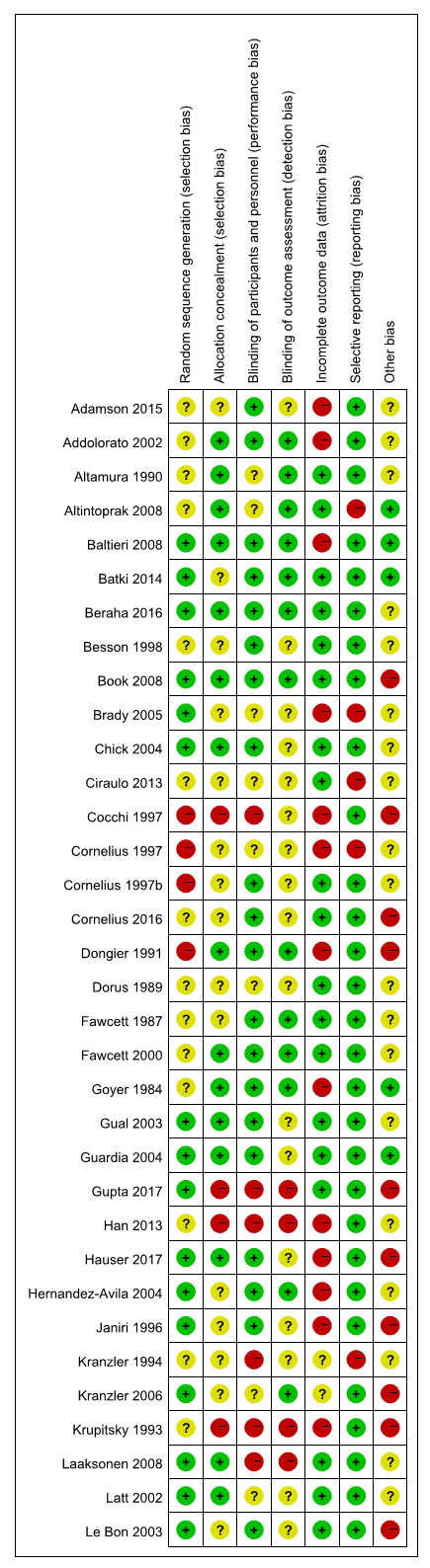

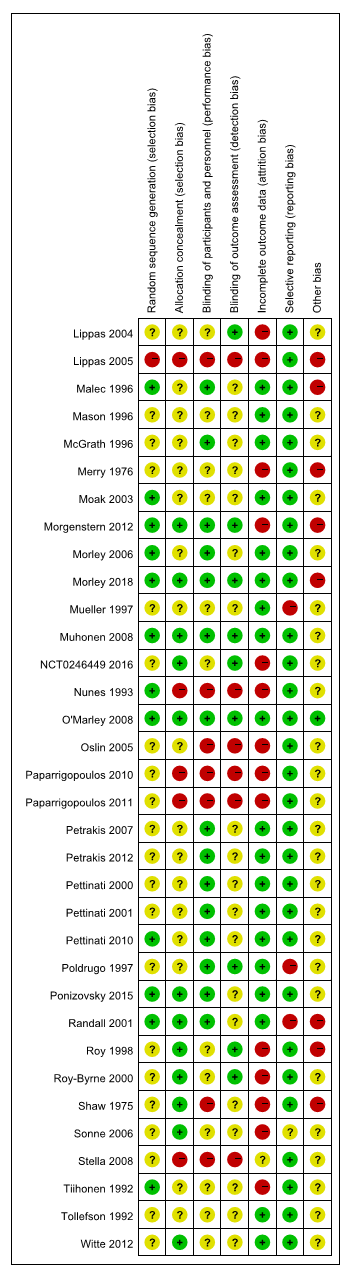


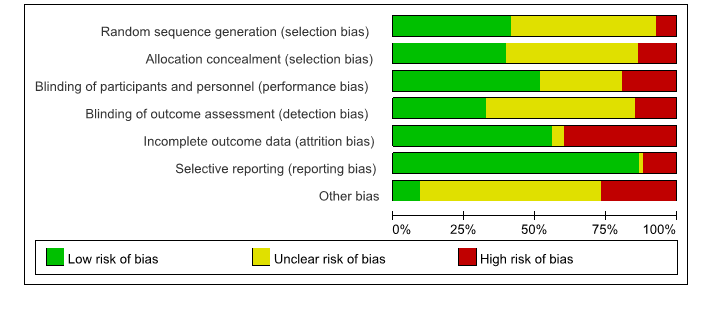


**Supplementary Figure 4B.** Risk of bias summary graph using Cochrane’s risk of bias assessment tool.
